# Strategies for managing spring frost risks in orchards: effectiveness and conditionality—a systematic review

**DOI:** 10.1186/s13750-022-00281-z

**Published:** 2022-09-01

**Authors:** Bianca Drepper, Brecht Bamps, Anne Gobin, Jos Van Orshoven

**Affiliations:** 1 Division Forest Nature and Landscape, University of Leuven, 3001 Louvain, Belgium; 2 Vlaamse Instelling voor Technologisch Onderzoek NV, 2400 Mol, Belgium

**Keywords:** Fruit tree, Damage, Prevention, Protection, Spring, Freeze

## Abstract

**Background:**

Spring frosts pose an important threat to orchard productivity in temperate zones and projections do not exclude damaging events in the future. However, there is no up-to-date and systematic comparison of the effectiveness and conditionality of the existing passive and active damage prevention strategies. This systematic review seeks to answer the questions “How do the performances of spring frost damage reduction strategies in temperate fruit orchards compare?” and “How do environmental conditions affect the effectiveness of frost damage reduction strategies in temperate fruit orchards?”.

**Methods:**

This review covers a large range of on-field strategies for the protection of flowering orchards against damage inflicted by late spring frost. All major temperate fruit tree crops and grapevines were included, provided that the performance of frost damage reduction was compared against a control in terms of bud and flower survival, yield and delays in flowering time, or ambient temperature change. Articles and reports were collected between June and October 2021 from the Web of Science Core Collection and regional indexes and from the databases Scopus, FAO AGRIS, USDA Agricola, CAB Abstracts and the Groenekennis database of the University of Wageningen, the Netherlands, as well as from relevant institutional websites and the Chinese scholarly search engine ‘Baidu’. Biases resulting from inadequate randomisation, incomplete reporting or deficient study designs were reported. Temporal and spatial research trends and gaps were mapped based on 104 selected studies (from 8970 identified studies). Data was extracted for every experiment that an article reported on, leading to 971 data points. Groups of frost protection methods were compared in terms of effectiveness whereby environmental factors were examined to explain the variation of the effectiveness by means of mixed linear models.

**Review findings:**

Most included studies originate from the United States and Europe more than from the temperate fruit production regions in Asia. An increase over time in the research on foliar applications, including growth regulation hormones was observed. Apple, peach and more recently grapevine were the most researched fruit types, followed by cherry and pear. The validity of the selected studies was generally low as measures of variability were reported only occasionally. Therefore, only descriptive comparisons of effectiveness were undertaken between intervention classes by fruit types. Sprinkler systems were found to perform best for most studied outcomes, while the emerging biochemical solutions revealed mixed results. The performances of resource-intensive heating systems did not outperform low-resource techniques such as tunnels or coverings of individual buds. The lack of reporting standards did not allow extensive correlations with ambient factors and reduced the transferability of the review’s findings. A need for standard protocols for experiments and reporting is therefore apparent.

**Conclusions:**

In this field, strong shortcomings in the documentation of experimental setups and reporting standards were exposed. Implications for policy making are limited while for research recommendations to reduce bias and increase comparability are put forward.

**Supplementary Information:**

The online version contains supplementary material available at 10.1186/s13750-022-00281-z.

## Background

In temperate fruit orchards, spring frosts are among the most important causes of yield reduction [1]. During the winter months, trees are in a dormant stage, without vegetative growth and with high tolerance against low ambient temperatures [2]. During the spring months, and throughout the plant development stages, the reproductive organs situated in the flowers are sensitive to temperatures below defined frost thresholds [3]. A single frost night on 19^th^ April 2017 led to 24% less apple and 12% less pear production in Europe [4] and frost in 2021 lead to damages of 24–30% in French vineyards [5].

In the context of a warming climate, there is agreement that in western and eastern Europe, Australia and South Africa flowering of pome, stone and vine fruit starts earlier [6–10]. However, also the last day of frost in spring occurs earlier [11–14], resulting in regional studies with contrasting conclusions about the evolution of frost hazard during flowering. Over the past decades, frequency and severity of frosts after bud break decrease in the United States and increase in Europe and Asia [15]. For smaller regions, disagreement persists in the literature, due to heterogeneity in the applied phenological models, climate models and assessment methodologies [11]. The resulting hypothesis is that despite the warming climate there is no evidence that frost will be of no concern in the future and preventing frost damage is expected to remain a major challenge for fruit producers.

There are already several overviews of regional guidelines for frost protection in orchards [16–21]. The current most prominent and extensive handbook was published by FAO and dates from 2005 [1]. An updated and systematic review seems needed to summarize new research findings and identify remaining research gaps. Since 2005 little work was done to review more recent prevention technologies such as wind turbines [17], sensor networks for early frost detection [22], foliar applications [23] and improved sprinkling systems. To our knowledge, the effectiveness of these and other damage prevention and mitigation methods is yet to be compared in a systematic manner and differences remain to be analysed in light of environmental conditions.

A substantial part of the published research dates from a time with low concern about consumption of fossil fuels, while more recent research focuses on resource-efficient ways of reducing frost damage, including low-discharge sprinkling systems and application of biochemicals to the trees. However, there is no objective comparison between a large set of interventions and their individual or combined effectiveness. Combined approaches have a high potential, for example, the combination of heaters and fans perform better than only heaters in Spain [24].

Effect modifiers are numerous in this research domain. The severity of damage by low temperatures is dependent on the phenological stage, the kind of fruit, the cultivar and the rootstock of the productive cultivar [25]. For apple trees, also the texture and related thermal capacity of the soil influences the damage, where sand or gravel is more susceptible than soils with finer texture [25]. Evidence on the influence of the surrounding environment is scarce but for several decades already there are indications that the nearby presence of lakes [26, 27] and forests [26] attenuate the frost damage. Topographic slope and aspect influence microclimates and potential for frost [28] and are associated with temperature gradients of several degrees. In addition, the effectiveness of wind machines reduces over inclined land by 0.28 °C per meter downslope from the tower on which it is located [20].

Several factors influence the temperature recordings: the humidity of the ambient air, whether it is shielded from radiation, the height above the ground, its technical accuracy, as well as the size of the sensor. Long and thin sensors close to tree branches are used to imitate flower styles and record considerably different temperatures compared to those measured by common weather stations: depending on the wind speed, the temperature of buds was commonly 1–2 °C lower than the air temperature [29, 30].

Techniques aimed at air mixing (e.g., wind turbines or helicopters) rely on temperature inversion, with a strong temperature gradient between a colder ground level and warmer higher levels (10–15 m). In windless conditions, the gradient can be up to 6.7 °C and around 3.3 to 4.4 °C at wind speeds around 0.9 m/s. No inversion is observed at wind speeds of 3.6 m/s and above [31, 32].

This review has been addressed as part of a research project on climate change-related frost damage in flowering orchards in Belgium and in cooperation with the Frost Inno project team of the Research Centre ‘PC Fruit vzw’ in Kerkom, Sint Truiden, Belgium. In the latter project, established methods and innovative new techniques were discussed and tested in consultation with a stakeholder group consisting of fruit farmers, researchers and representatives of the frost protection and monitoring industry [33]. Expertise from the research centre directly shaped the review design. The review also addressed the main need formulated by an EIP-AGRI Focus Group on frost protection in fruit orchards, consisting of stakeholders and experts from across Europe [34]. The top three research needs that were mentioned were (i) “Studying and comparing the effectiveness of methods under different conditions”, (ii) “More biology to the models” and (iii) “Establishing a database on potential yields for different species/varieties and critical temperatures at species/variety level” (p. 35).

## Objective of the review

This review aims to answer two research questions: *“*How do the performances of spring frost damage reduction strategies in temperate fruit orchards compare*?”* and “'How do environmental conditions affect the effectiveness of frost damage reduction strategies in temperate fruit orchards?”.

The main objective was to compare the effectiveness of interventions that target the (partial) reduction of yield losses due to spring frost. To support informed decision making, the aim was to identify favourable or unfavourable environmental conditions for a class of interventions. However, it emerged from the studies that the reported environmental conditions at the studied orchards were not sufficiently documented. The correlation is thus limited to the study’s meta-data or information that could be extracted from external data sources. To underline the need for more detailed description of environmental conditions, an overview of (un)available information on effect-modifying factors is given.

## Methods

### Deviation from the protocol

In contrast to the methodology described in the protocol [35], fewer data sources were consulted. While all specialized databases and websites were queried as described in the protocol, additional searches were limited to the scholarly section of the Chinese search engine ‘Baidu’. The American search engine ‘Google Scholar’ was not used because the underlying algorithm does not return replicable search results [36]. To find grey literature, priority was given to specialist websites and databases.

For the same reason, no ‘snow balling’ was done, i.e. no articles were identified by examining the references used in the detected ones. For data extraction, options for pre-coded variables were added. The category ‘external validity’ was removed from the validity judgement as it appeared to reflect the study setup only (field or lab). Publication biases were not examined as the included articles did not report the variances in their results.

Efforts to contact corresponding authors were limited given the generally early publication years.

Instead of creating an online evidence atlas, the spatial and temporal distribution of research by fruit and interventions is presented in maps and heatmaps.

### Search for articles

#### Search terms and strings

All databases were searched using the search strings specified in Additional file 1: Table S1. They contain a list of population descriptor terms (singular, plural and Latin expressions) and a term for conditions of below-zero temperatures or the relevant phenological phase. An example string (for the Web of Science) is the following:

TS = ( ( "orchard$" OR "fruit tree$" OR "pome fruit$" OR "stone fruit$" OR "hesperidium" OR "hesperidia" OR malus* OR pyrus* OR prunus* OR persea* OR citrus* OR "vitis vinifera" OR "apple$" OR "pear" OR "pears" OR "cherry" OR "cherries" OR "peach" OR "peaches" OR "nectarine$" OR "plum" OR "plums" OR "apricot$" OR "avocado$" OR "lemon$" OR "orange$" OR "grapefruit$" OR "mandarine$" OR "pomelo$" OR "grape$" OR "vine$" OR "vineyard$")

AND

(((prevent* OR protect* OR "manage" OR "management" OR damag* OR injur* OR “flowering" OR "flowers" OR "bloom" OR "blooming" OR "blossom*") AND ("frost" OR "frosts" OR freez* OR "cold weather")) OR ((("cold” OR "low* temperature*" ) NEAR (damag* OR injur*)) OR "Freeze avoidance" OR "antifreeze" OR "anti freeze" )) )

#### Search sources

Following the protocol [35] the most relevant international databases for academic literature and more specialized or regional literature databases for the agricultural domain were searched: Web of Science (including the Core Collection and Chinese, Korean, Russian and Latin American Indexes), Scopus, CAB Abstracts, Agricola (USDS National Agricultural Library), Agris (Food and Agriculture Organisation of the United Nations) and Groene Kennis (Wageningen University, the Netherlands), see Table 1. Additionally, and to cover the Asian continent, simplified searches were conducted in ‘Baidu scholar’, from where additional relevant studies were added to the review.

**Table 1:** Overview of databases and libraries

Platform/publisher	Database/library	Index/specification	Years covered	Hits on 15/06/2021
Web of science	Core collection	SCI-EXPANDED	1955–2021	2475
		SSCI	1956–2021	
		A&HCI	1975–2021	
		CPCI-S	1990–2021	
		CPCI-SSH	1990–2021	
		BKCI-S	2005–2021	
		BKCI-SSH	2005–2021	
		ESCI	2005–2021	
	CSCD		1989–2021	415
	KJD		1980–2021	83
	RSCI		2005–2021	50
	SCIELO		2002–2021	58
Elsevier	Scopus	‘Documents’	1788–2021	2518
FAO	AGRIS	‘Publications’	1954–2021	1510
USDA National Agricultural Library Agricola	NAL Article Citation Database, NAL Cataloging Database		1905–2021	1123
CAB Direct	CAB Abstracts		1910–2021	
WUR Groenekennis	All content		1543–2021 1981–2015 (tags)	63 (EN) 44 (DE) 238 (NL) 78 (tags)
Baidu Scholar				230 (Hits on 27/09/2021)

In addition, specialist websites of major international and national institutions (selected based on expert recommendations) were searched for generic words (Frost, freeze, gélée, heladas, vorst) or combined terms (("gélées" OR “gel”) AND ("protection", OR "proteger")). The list is identical to the list presented in the protocol [35]. Changes with the search strings presented in the review protocol concern the search in the scholarly division of the search engine ‘Baidu’ and are highlighted in Additional file 1: Table S1.

#### Search limitations

Searches are conducted in English in all databases and search engines other than the Groene Kennis Database, as the content of this database was indexed with Dutch and not with English keywords. The earliest retrievable records of any queried library or database were retained in Table 1 for full transparency. No date imitations were used. Searches were first conducted in June 2021.

With the access to full texts from one (European) university alone, not all identified research could be used. If this review is to be updated, more studies could be included.

#### Estimating the comprehensiveness of the search

The most relevant references in terms of recent and complete reviews (were used to define necessary search terms during the creation of the protocol. We did not compile a clear list of key references to be returned as no objective list could be created. Alternatively, we compared the search results with the relevant papers on the first four pages of google scholar search for (“frost” AND “protection”). All relevant studies were returned by the review with one exception. This study would be excluded at the full text level, as the results were not reported in any of the outcome categories.

#### Search results

Articles returned by the searches were registered in the EndNote software and duplicates were removed following a protocolized sequence suggested by Bramer et al. [37].

The included references were gathered in the reference manager ‘Zotero’ from where full-text retrieval was organised. Efforts were made to contact authors and local archives were visited but hard copies dating from before the 1980s appeared not available in these archives and could not be found elsewhere.

### Article screening and study eligibility criteria

#### Screening process

The collected and deduplicated references were uploaded to ‘Rayyan’, an online tool for screening articles based on title, abstract and metadata [38]. Information on the language of the full text was used to discard articles in any language other than English, French, Spanish, German or Dutch.

The Rayyan interface allows filtering entries by keyword, which was used to remove ineligible studies. However, all titles and abstracts were read prior to removal. This way articles on plants other than those mentioned in Table 2 were excluded. Finally, reviews, model developments and surveys were excluded. Simultaneously the remaining criteria (Table 2) were assessed, article by article.

**Table 2:** Primary inclusion and exclusion criteria based on title and abstract

Criteria	Inclusion	Exclusion
Population	All temperate perennial fruit trees of commercial interest (Apple, Pear, Sweet cherry, Peach, Nectarine, Plum, Apricot, Avocado, Lemon, Orange, Grapefruit, Mandarin, Pomelo, Grapevine)	Low-height shrubs Berry plants Other non-fruit horticulture Other non-temperate horticulture
Interventions	Physical on-site treatments or devices that can be applied in anticipation, during or just after a frost event, like wind machines, sprinklers, foliar applications of chemicals, coverages, modified pruning	Management choices like income or crop diversification Crop breeding Genetic modifications Financial insurances
Outcome	Measures of ambient temperature Measures of damage to flowers, buds or fruitlets Measures of production Advance/delay of phenological stages	Evolution of frost hazards or vulnerability through time and space Evidence on post-harvest conditions Change in cold hardiness during dormancy
Comparators	Investigations of effectiveness against a control population or variants of the same treatment/device	Lack of comparators or other measures of success Model vs observation
Climate zones	Temperate Mediterranean	Tropical Subtropical Cold climates
Language	English, French, German, Dutch, Spanish	Any other language
Type of publication	Peer-reviewed journal Organisational report Professional journal article Thesis	Web page/Blog Unpublished communication
Type of study	Field experiment Greenhouse experiment Laboratory experiment	Literature review Mathematical model Risk assessment studies Micro meteorological studies to explore the potential for an intervention to function without observations of the intervention itself (i.e., Inversion strength characterisations)

The first author of the review paper screened 88.5% of the articles while the second author dealt with the remaining 11.5%. A total of 7.0% of the articles (Title and abstract: 416/5950) were screened by both authors independently from each other’s judgements. The limited number of conflicting interpretations (5.5% of the 416 studies) between the two screeners were recorded and reported in Additional file 2. Conflicts were discussed among the authors and in case of doubt the article was included for further processing. That is why a relatively large share of articles was discarded later, i.c. at the data extraction stage when the full text was read. For full text screening, 20 articles (7.0%) were assessed by both authors independently from one another, of which 8 were included. Reasons for exclusions are retained in Additional file 2. The extraction fields were adjusted to overcome identified sources of misinterpretation during the establishment of the protocol. During the process of the review, some fields were split to allow for more detailed recording and easier comparison. Validity assessment was first found to lead to conflicts until the description of the fields in sysrev was made more explicit and consistent. No author screened their own publications.

#### Eligibility criteria

The criteria for including or excluding articles are given in Table 2 and are unchanged compared to the protocol [35].

### Data coding and extraction strategy

The coding of the articles strictly followed the protocol except that for certain pre-coded variables (intervention class and temperature sensor position) additional options were added, to cover all encountered situations. Also, new fields to specify the (tree or vine) training system and tree heights were added. All coding options are detailed in Additional file 3. The majority of data was extracted on the ‘sysrev’ platform (www.sysrev.com) because of the convenient interface with dropdown menus for precoded variables and options for binary variables. Sysrev also comes with a capacity for multiple users to operate and solve conflicts. However, the interface has limitations for studies covering multiple experiments leading to different parameters in the precoded questions. It was therefore necessary to continue the extraction manually using a spreadsheet (‘Excel’). In this step, multiple experiments per article were separated into individual entries in the final database. The table with the extracted data is provided in Additional file 4. The consistency of the extraction of meta-data, qualitative and quantitative data as well as the validity assessment by the two responsible screeners was tested in a subset of seven publications (6.7%) during the protocol development. The extraction was consistent between both screeners. Authors were not contacted in case of missing data and articles were excluded at the full text level if no outcome matched the relevant outcome categories reported.

### Potential effect modifiers and reasons for heterogeneity

The second objective and the second research question are related to the potential effect modifiers, i.e. environmental conditions that explain variation of findings between studies. The list of effect modifiers was compiled together with experts from the Flemish fruit research centre PC Fruit and the literature. Soil texture, elevation, crop cover (leaf area), presence of ground covering crops, tree phenology as well as the age of the orchard influence the spatial temperature gradients [39]. Effects of row orientation and orography (aspect) on temperature could not be detected. Based on [38] and [25], a total of 13 potential effect modifiers was recorded: Height above sea level, landform (any indication of the terrain), surrounding land use types, dominant soil texture, rootstock, development stage [40], pruning and training schemes, wind speed and direction, maximum relative humidity, minimum ambient temperature, inversion strength, duration of the frost event and the type of groundcover between the tree rows. External data sources are used for height above sea level (global Shuttle Radar Topography Mission [41] and soil texture (SoilGrids [42], using the soilDB package in R [43]).

### Study validity assessment

Every article included was assessed for validity using the criteria in Table 3 as defined in the protocol, which were established in collaboration with the PC Fruit research centre. General bias criteria [44] refer to the scientific procedure: whether the studied samples/trees were randomly selected; whether steps were taken (i.e., blinding) to avoid subconscious over- or under-recording or to account for differences between researchers; and whether all results (experiments and individual data points) were presented. A second set of criteria intended to distinguish between specific study set-ups: The sample size should exceed defined minima (Table 3); the description of the populations should be comparable; temperatures should be measured locally, and results reported with a measure of variability. In experimental fields the control and intervention populations (i.e. rows of trees) might not have the same baseline conditions which might interfere with the effect sizes. Especially so, if the rows are on fields that are distant from each other and for example on different soils, or on lower/higher laying parts or wind shielded sides of a field. In practice, we assessed potential differences between parcels by their locations, which were usually mentioned, and between rows, if maps and images were provided.

**Table 3:** Study bias assessment categories

	Risk of Bias	High	Low
General Bias Based on Bilotta et al. [44]	Selection bias (Inadequate randomisation)	No randomisation (site /tree selection based on availability, expert judgement or results)	Randomisation (site /tree selection based on random number generator/tossing a coin)
	Performance and detection bias (researcher bias)	No blinding / Blinding broken / insufficient information	Blinding done
	Reporting bias (incomplete results (variables))	Not all pre-specified outcomes reported; measurements/methods for some outcomes were not pre-specified; incomplete reporting of one or more outcomes; not reporting on an expected outcome	Study protocol is available and pre-specified outcomes are reported on; study protocol not available but all expected outcomes are reported on
	Attrition bias (incomplete results (observations))	Resulting data points lower than expected from the methodology; No reason given for missing data related to outcome; bad imputation	Reported data points correspond to methodology; data omission or imputation well documented and justified
Adapted criteria	External validity [45]	Spatial replication (Experimental units) 2 field (widespread application) Or 2 rows (row-based installation) Or 2 trees (targeted application) OR Temporal replication 1 frost night (temperature change) 1 year (yield/damage) Observations (assessment level) per experimental unit < 4 trees (widespread or row-based) < 4 branches (targeted application)	> 2 fields > 2rows > 2 trees > 1 frost nights > 1 years > = 4 trees > = 4 branches
	Baseline of test and control groups compared	At least one of the following differs or is not described: management (pruning), soil type, topographic position, differences in cultivar and/or rootstock, orchard age	All of the following factors are comparable: management (pruning), soil type, topographic position, differences in cultivar and/or rootstock, orchard age
	Location of temperature sensor given	Closest meteorological station > 10 km away; no detail given	Local sensors on field and beside flowers at several tree heights (lower, middle and upper position in tree)
	Reporting method	Mean effects per experiment only	Raw data, Measure of variation

A general appraisal of validity was expressed by counting the criteria as follows: at most one high risk of bias leads to an overall high validity; three or more high risks of bias lead to an overall low validity. All remaining studies were rated to be of medium validity. In deviation from the protocol, the category label ‘External validity’ was given to the description of the former category ‘Number of spatial replications (experimental units) and observations’. The former category ‘External validity’ was removed from the validity judgement as it did not reflect more than the study setup (field experiments vs. controlled climate chambers) and hence did not contribute to assessing the risk of bias.

No studies were excluded based on these criteria, but the outcomes were plotted in different colours according to the overall risk of bias.

### Data synthesis and presentation

#### Classification of techniques

In order to address the primary research question *“*How do the performances of spring frost damage reduction strategies in temperate fruit orchards compare*?”* the encountered intervention techniques were grouped into 8 classes (Table 4).

**Table 4:** Classification of interventions

Class	Count	Included interventions
Water	17	Automated sprinklers; micro sprinklers; micro sprayers; evaporative cooling; overtree sprinkler; undertree sprinkler; flippers
Wind	11	Portable wind machines; basic and fuzzy controlled wind machines, sis; upward wind machines; double fans and single fans on towers
Covering (field)	5	Nettings; tunnels with/without openings
Heating	12	Heating cables; heating quilts; spot-scheu; gas heating systems, frostbuster; frostguard; oil heaters; candles
Cultivation practice	8	Early, mid, late pruning; pruning frozen branches; raking; removing cover crops; fumigation (heat and smoke); windbreaks; coal dust on bare strips; interplanting pecan or pine trees; herbicides
Foliar applications	43	Amigo oil (soybean); GA 4 + 7; GA3; GIBB plus; N-Propyl Dihydrojasmonate (PDJ); Promalin; Azolon; ascorbic Acid (vitamin C); Naphthaleneacetic Acid; NPK; Alpha Tocopherol (Vitamin E); Frostgard; Peat Extract; Etephon; Urea Spray; Paclobutrazol; Teric Acid; Dormex; Regalis; Semperfresh; Protone; Frostshield; Methyl Jasmonate; Salicylic Acid; Systhane; Amid-Thin
Combined approaches	5	Heated tunnels; sprinklers under screens; hot water underneath wind machines; sprinkling hot water
Covering (buds)	3	Cellulose nanocrystals; cotton candy with straw; polyethylene bags; sawdust

#### Descriptive analysis of the research interests

The spatial and temporal patterns and trends were geo-referenced and further aggregated by country to allow for spatial research trend analysis. Research gaps were investigated in terms of publications by fruit type, intervention category and year. As anticipated in the protocol, it became apparent that only 9 of the 104 included studies reported a measure of variability (e.g. variance) of the effectiveness of the studied damage prevention interventions. Among these, the sample size was not always disclosed. Therefore, it was not possible to conduct a full meta-analysis of the effects nor to quantify heterogeneity between studies. The total of 796 experiments for which mean effects on one of the outcome categories were reported, allowed however to compare effect sizes in terms of Raw Mean Difference (or ratios in the case of yield values) between intervention classes and between fruit classes by means of categorical swarm graphs and indicative statistical testing.

#### Effect size calculation

Effect sizes were calculated as X_intervention_ – X_control_ for all outcomes but yields as defined in the protocol. One modification to the protocol was made regarding the definition of outcomes, such that a higher effect size consistently implies higher effectiveness. Instead of the (flower or bud) ‘damage rates’ defined in the protocol, the (flower or bud) ‘survival rates’ were recorded/calculated from the reported study findings. Thereby the survival rate is 100% minus the damage rate. Reported raw mean differences between the control population and the treated population in terms of temperature [°C] and delays in budburst or flowering [days] were recorded without further transformation other than converting degrees Fahrenheit to degrees Celsius. Reports on yields required conversions to a common metric (kg/tree). Yields per hectare were divided by reported or estimated numbers of trees.

These estimations are based on the reported row and tree distances, field sizes and the simplification of assuming squared fields. We have divided the square root of the field size by the row and by the planting distances, and multiplied the resulting number of trees by the resulting number of rows. We further assumed no space between the rows and the edges of the field.

To ensure comparability between orchards and vineyards, yield ratios were calculated instead of absolute differences. The natural logarithm of the ratio was then computed to continue with a scaled result, where 0 means no change.

All treatments within one article were regarded in the same way in the meta-analysis whilst these treatments were not always hypothesized to have equally beneficial outcomes by the authors of the individual studies. For example, often the concentration of the active compounds in foliar sprays or the discharge applied by water spraying systems were assessed at extremely low and detrimental high levels. No attempt was made here to distinguish between effective and ineffective parameters due to a lack of objective information.

For a meta-analysis of continuous outcome variables, information on the mean and either the standard deviation, variance or standard error for each treatment group is needed [46]. Studies for which this information is missing must either be excluded from the meta-analysis or alternative methods must be used to derive an estimate of the mean and its associated measure of variability. Multiple methods exist to estimate mean values from other summary statistics (e.g. median, quantiles…) and standard deviations from the sample size or range. Besides missing measures of variability, information on sample sizes, p-values, summary statistics etc. were in most cases also missing, unclearly reported or inconsistently defined between the studies. Therefore, we refrained from presenting our results as if they were the result of a statistically sound meta-analysis.

#### Testing for differences between interventions and fruit types

Shapiro–Wilk tests indicated significant deviation from the normal distribution for all four outcome variables (flower or bud damage, temperature change, yields and delay of budding or flowering). Therefore, non-parametric Kruskal–Wallis rank sum tests were applied to test for significant differences between intervention classes and between fruit classes respectively for the different outcome variables. When a significant difference between the categories (classes of interventions or fruit classes) was detected, pairwise Wilcoxon tests allowed to test for pairwise differences between all considered categories. For the Wilcoxon tests, the p-value adjustment method was set to “BH” (Benjamini-Hochberg) in order to reduce misclassification errors [47]. All statistical testing was done in R software package ‘stats 3.6.2’ [48]. However, these statistics are merely indicative, provided the unknown precision.

#### Mixed linear models

For the dependent variables that were defined in the protocol, mixed linear models were applied to detect the effects of ambient characteristics on the effectiveness of a given intervention. These dependent variables include the recorded minimum temperature during the frost, the soil texture (approximated by the % sand in the topmost layer, as derived from the SoilGrids Database [42, 43]), the elevation and the latitude (in absolute numbers). Random factors include the intervention class, the fruit type and the development stage during which frost occurred. Every model was run separately for every outcome variable (flower or bud damage, temperature change, yields and delayed budding or flowering). In accordance with the protocol a general linear model was defined covering the varying numbers of experiments for which the required observations were available. Interaction terms were specified with ‘:’. However, also these statistics are merely indicative.

$$\left(\text{Model} \,1\right)\, outcome\sim\,elevation+absolute\,latitude+elevation:absolute\,latitude+\%\, sand\, in\, toplayer+minimum\,temperature$$

To control for random factors the following mixed-effect linear effect models were constructed using the lme4 package (version 1.1.27.1) [49] in R:

$$\left(\text{Model}\, 2\right)\, outcome\sim\,elevation + absolute\,latitude + elevation:absolute\,latitude +\%\,sand\,in\,toplayer + minimum\,temperature +(1| Intervention)$$

$$\left(\text{Model}\, 3\right)\, outcome\sim\,elevation + absolute\,latitude + elevation:absolute\,latitude +\%\,sand\,in\,toplayer + minimum\,temperature +(1| Fruit\,class)$$

$$\left(\text{Model}\, 4\right)\, outcome\sim\,elevation + absolute\,latitude + elevation:absolute\,latitude +\%\,sand\,in\,toplayer + minimum\,temperature +\left(1\right| Development\,stage)$$

## Review findings

### Review descriptive statistics

**Fig. 1:**
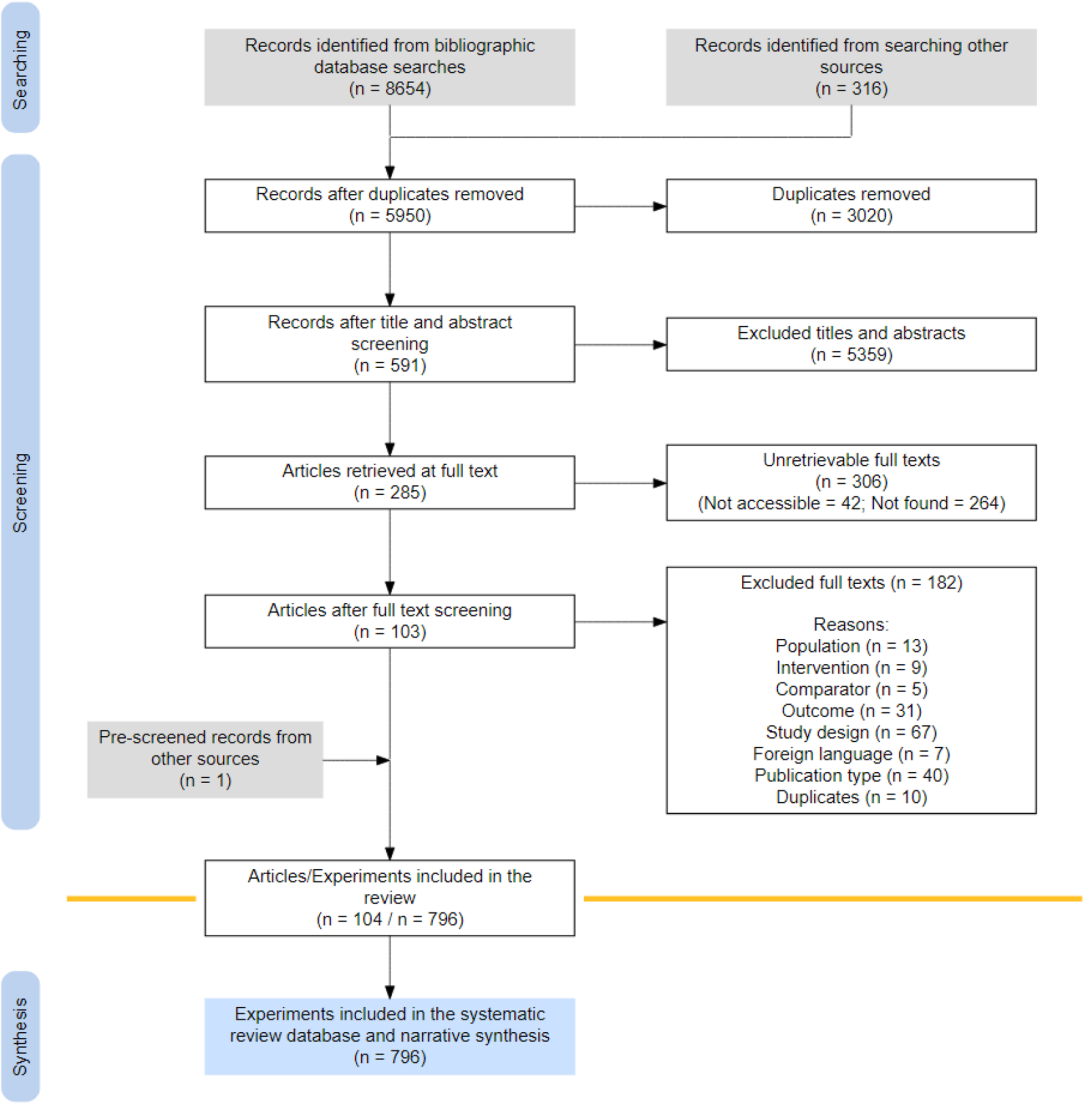
Reporting standards for Systematic Evidence Syntheses (ROSES) flow chart of the article selection process showing literature sources and inclusion/exclusion of eligible studies. The 104 included articles contained results of 796 experiments

From searches conducted between June and September 2021, a total of 8655 articles were returned from the queried databases and search engines (Fig. 1). After deduplication, 5950 articles remained of which title, abstract and metadata were checked for inclusion. This step was done by two authors using the Rayyan web tool. Among the 416 articles screened by two authors (7.0%), there were only 24 conflicting in- or exclusion decisions. In Additional file 2, a table of all references with their reason for exclusion is given. The main reasons of exclusion were a non-relevant topic (n = 1718), a non-relevant population (n = 976) and a language other than English, German, French, Spanish or Dutch (n = 840). The R software package ‘ROSES_flowchart 0.0.1’ was used to create the ROSES flow chart depicted in Fig. 1 [50].

A subset of 591 articles were retained as eligible, see Additional file 5 (containing a table of all eligible articles mentioning the database where an article was found). However, only 281 full texts were retrieved, of which 178 were discarded after reading the full text. The main reason here were research outcomes outside of those eligible for the review. The unretrievable articles are listed in Additional file 6

In conclusion, 104 articles were included for the analysis. Among the 104 included articles, 92.3% reported on more than one experiment (796 in total). With regard to the reported outcome, 16.5% of the articles reported on more than one outcome measure per study, which were individually retained as 971 effect sizes in the final dataset (Additional file 4).

**Fig. 2:**
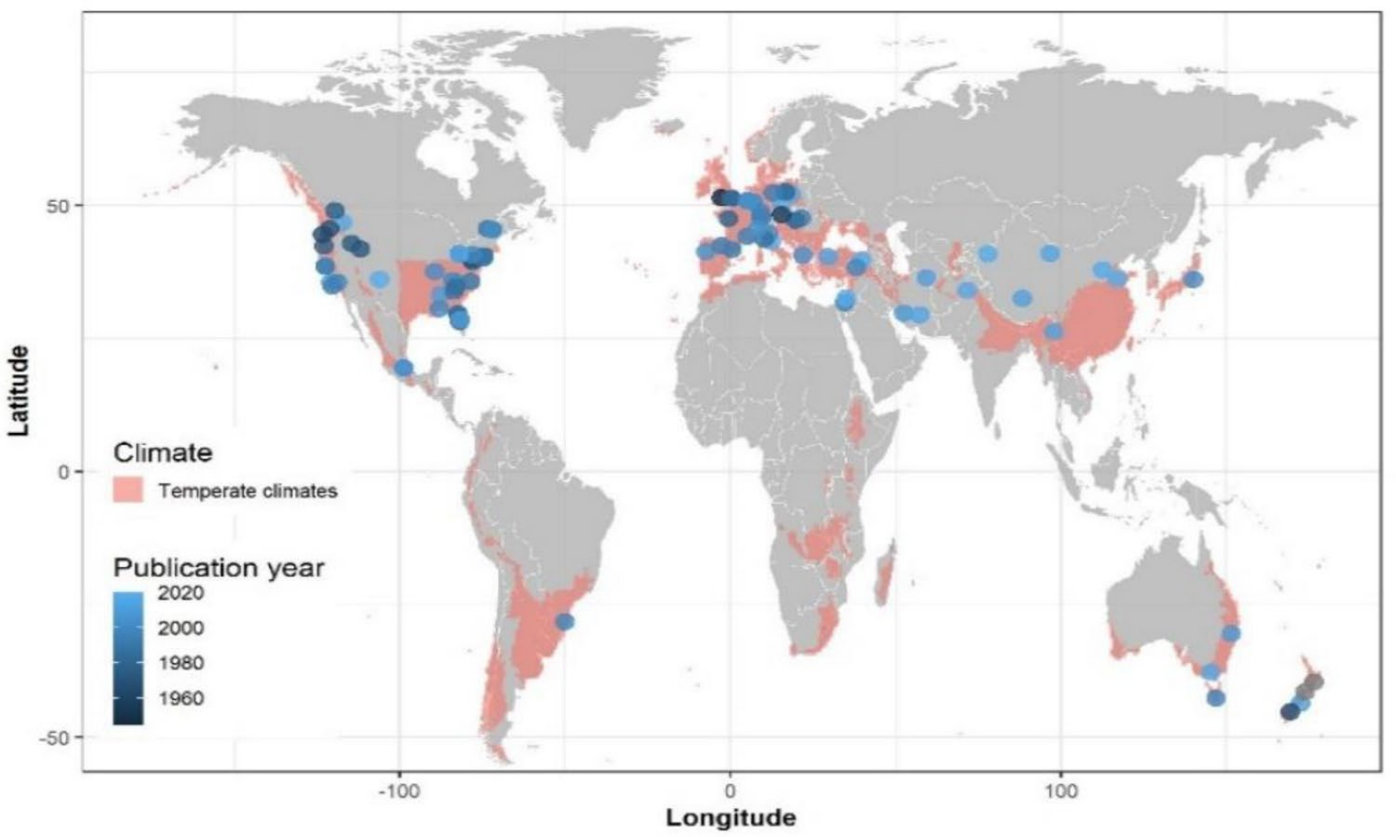
Geographic distribution of the included studies within zones classified as ‘temperate’ according to the Köppen-Geiger system (type ‘C’)

Despite efforts to include literature from Asia and Latin America, 84.6% of the results were confined to North America, Europe and Oceania (Fig. 2).

**Fig. 3:**
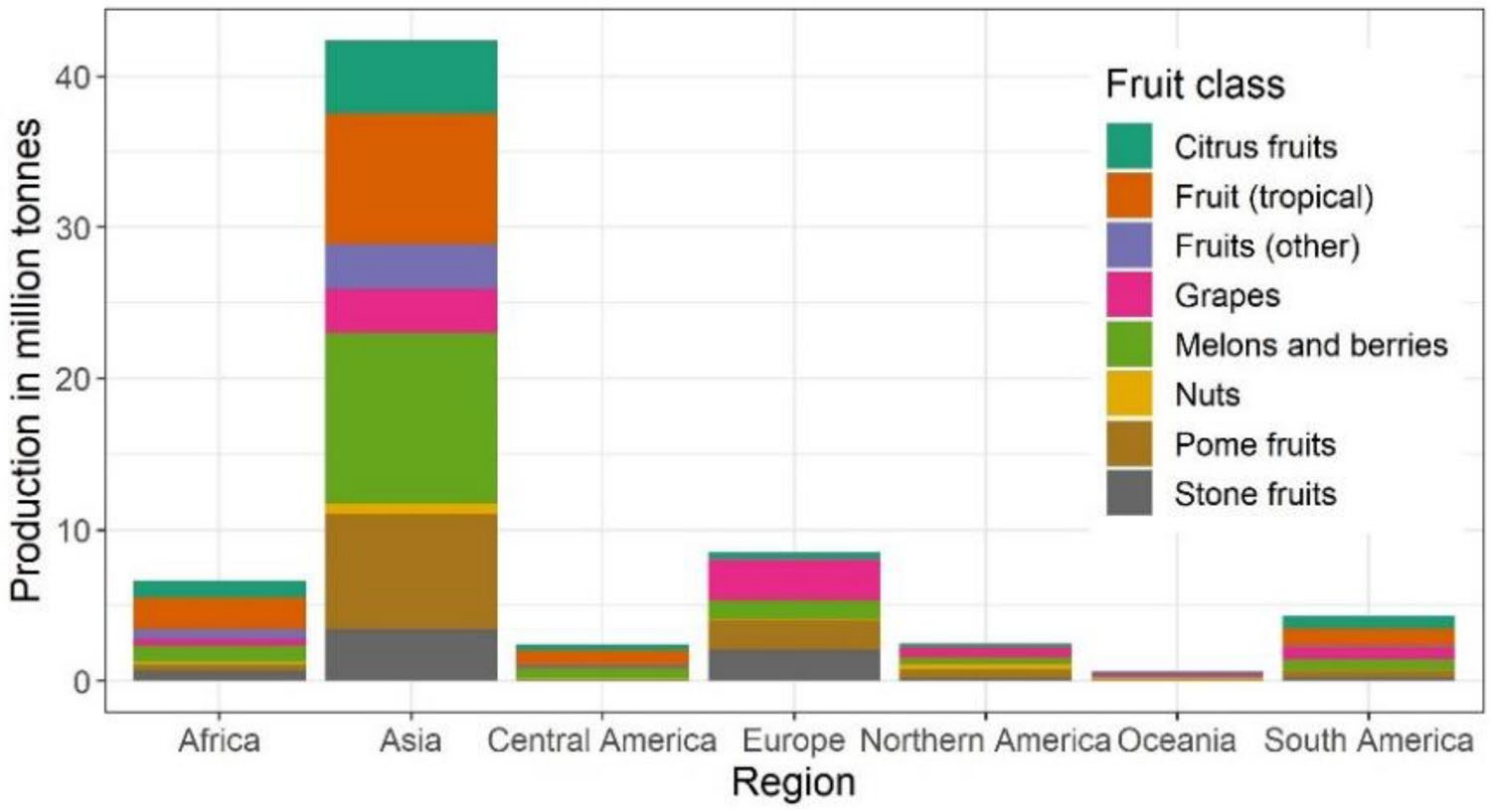
Production of fruit in 2018 in tonnes by continent and fruit type [51]

No study from the African continent was considered nor for the mountainous parts of south-east Asia. Relatively important producing countries like Argentina and Chile do not appear. Studies from these countries were initially detected but did not fulfil the inclusion criteria in Table 2. Therefore, the most important producing regions in terms of harvest weight are severely underrepresented and the dominance of studies from North America is not in accordance with the global distribution of annual total fruit production (Fig. 3).

**Fig. 4:**
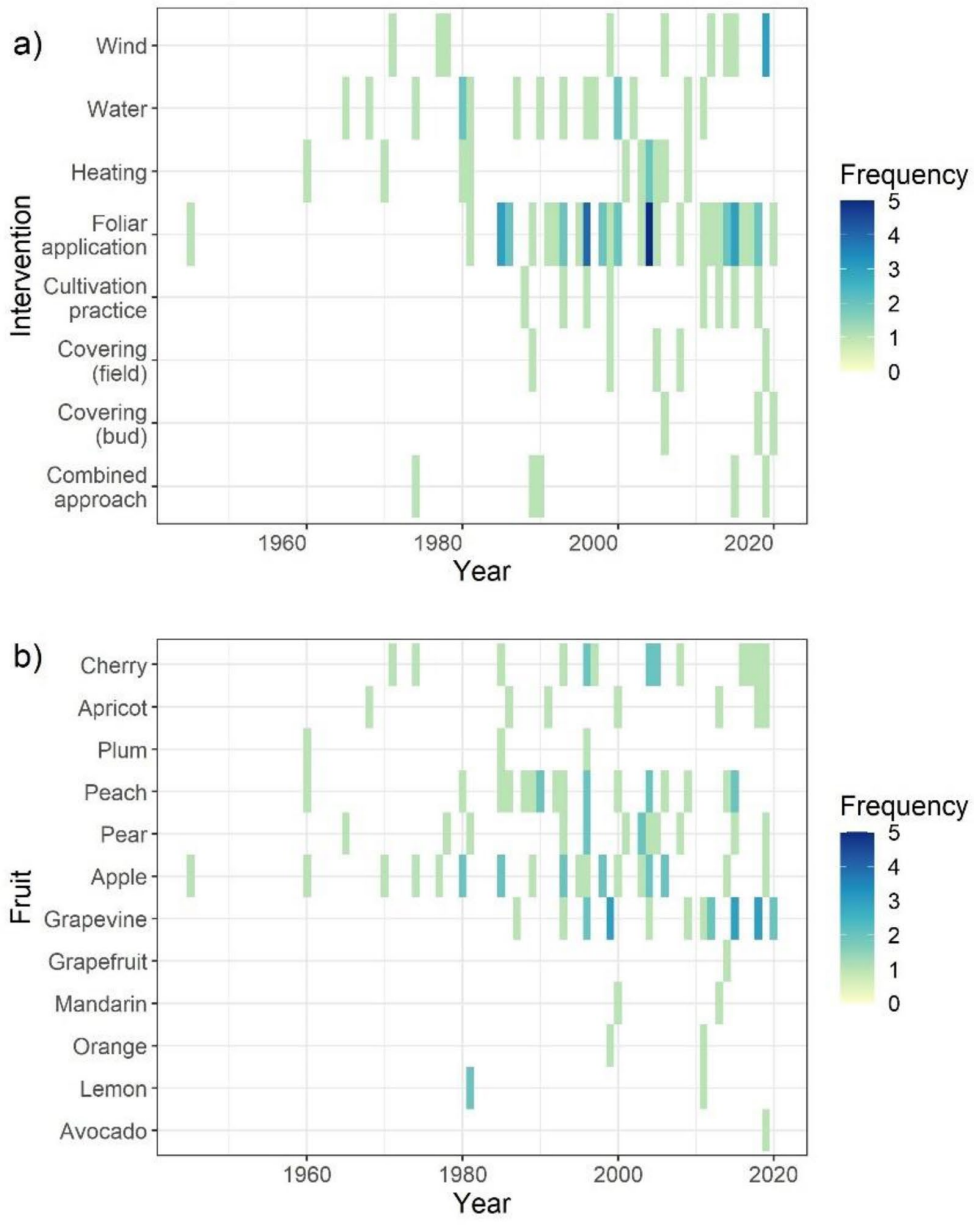
Timeline and frequency of included studies by type of intervention and type of fruit (based on individual studies)

The body of included literature spans a period of 1905 to 2021 with the oldest publications coming from North America and England (Fig. 2). During this period, there was a constant release of publications dealing with apple while in recent years, grapevine gained increased attention (Fig. 4b). Historical regionally destructive frost episodes, like the ones in 1991 and 2017 in Europe, did not show in the data. The studies of peach, cherry and apple were spread over countries and continents, while other fruits were confined to certain regions, e.g., pear in Western Europe and citrus to the United States. The latter is overrepresented in the dataset since nearly all included fruits are researched in this sub-continent.

**Fig. 5:**
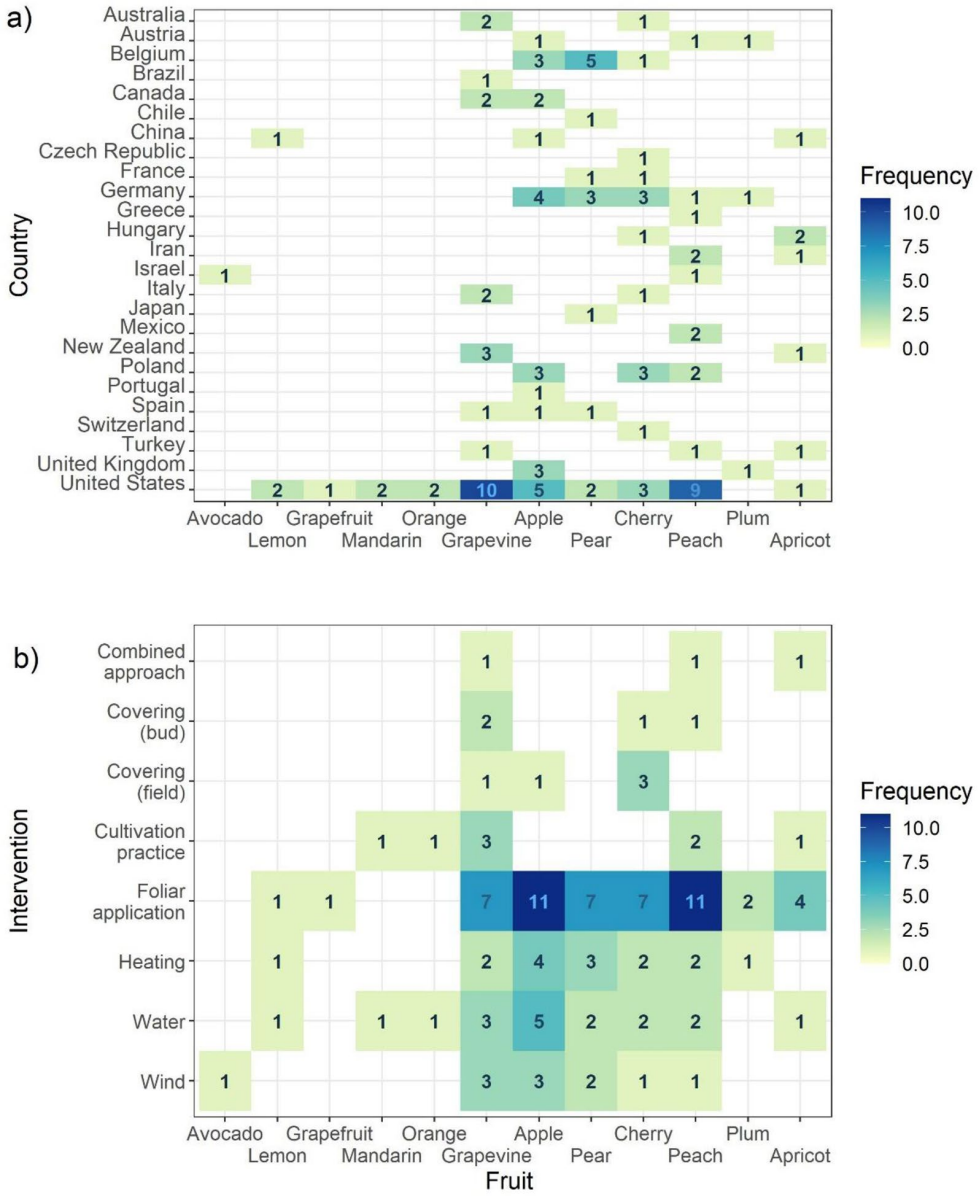
Matrix of studied fruit by country (**a**) and by intervention class (**b**) (based on individual studies)

Temperate fruits in this review also include citrus fruits [52], for which numerous studies on frost hardiness and leave or stem survival (during the winter months) have been published. Since in this review, outcome measures were restricted to effects on buds, flowers and yields, in relation to spring frosts, the majority of these citrus fruit studies were excluded. Citrus fruit (lemon, orange, grapefruit and mandarin), as well as avocado, are therefore underrepresented in Fig. 4b and in Fig. 5a, b.

Interventions based on water, wind or heating installations were continuously studied through time (Fig. 4a). With exception of the oldest included study, the interventions grouped as ‘foliar applications’ constitute a comparatively recent subject of research interest. Alternative approaches, including covering of the buds, rows or entire orchards, as well as cultivation practices (e.g., pruning or mowing), also received more attention in the last decades. Not any intervention type was studied for all included fruits (Fig. 5b). Foliar sprays were mostly studied in relation to apple, peach, cherry and pear. Wind machines were relatively often studied for vineyards.

### Narrative synthesis including study validity assessment

**Fig. 6:**
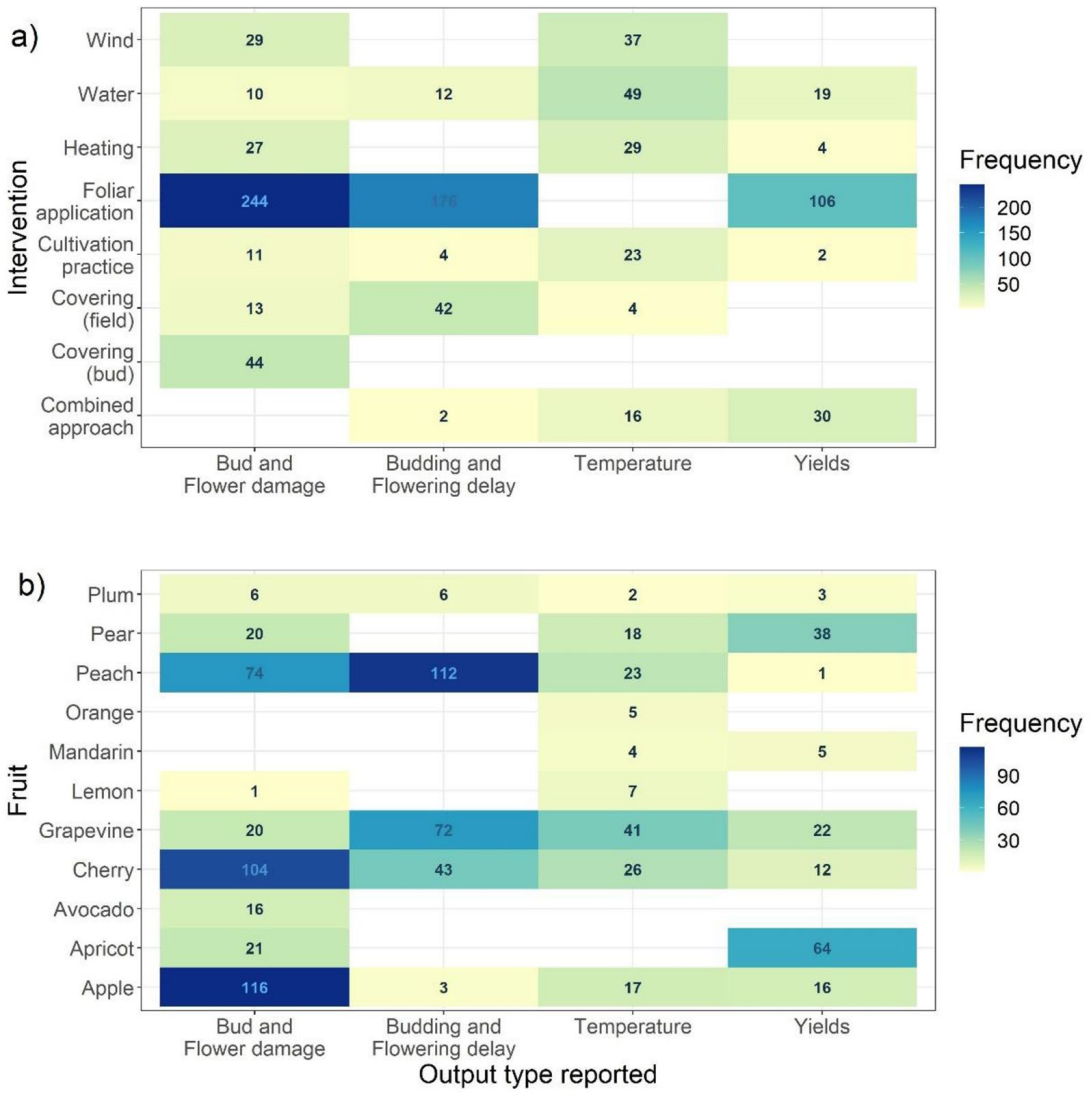
Matrix of output type by intervention class (**a**) and studied fruit type (**b**) (based on individual experimental outcomes (n = 971))

In the 104 selected articles, 796 studies or experiments were identified which yielded 971 data points on effect sizes (Fig. 6). The most common outcome was bud and flower damage reduction. Data extracted from each study, including metadata and individual study findings, along with other key information such as study location and reporting of effect modifiers are accessible in Additional file 6.

**Fig. 7:**
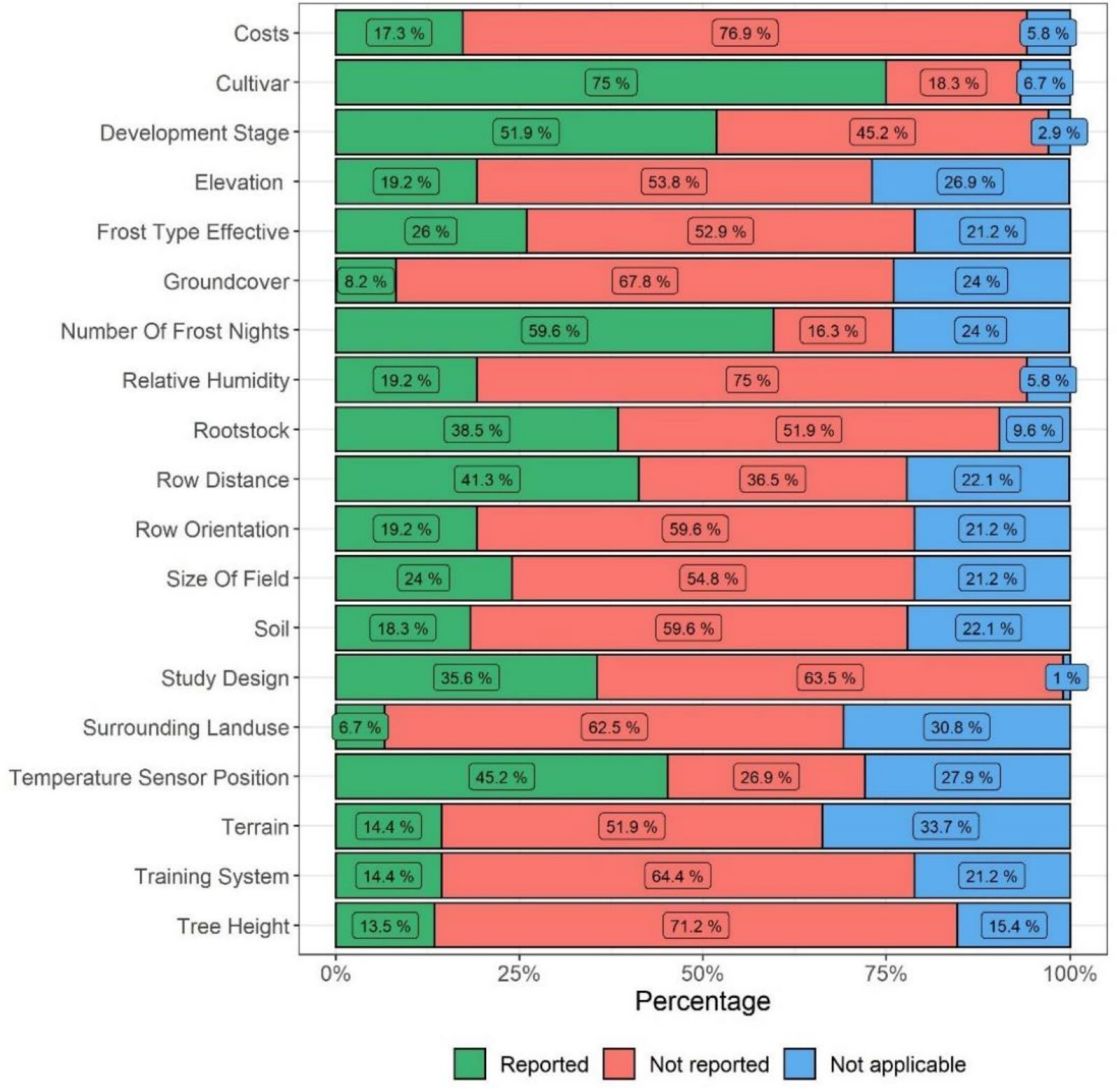
Mentioned effect modifiers and environmental conditions in the studied articles

Figure 7 shows the share of studies that report on selected details. Most studies reported on the cultivars (75%), but only 38.5% on the rootstock, despite the strong influence of the latter on frost resistance [25]. Only few studies reported on the landform or terrain of the studied fields (14.4%) and 6.7% reported on notable surrounding land use, such as the presence of waterbodies and the ground cover between the rows. The mention of pruning and training schemes is as low as 14% although they determine the amount of 1-year- and multi-year wood and the resulting flowering times and exposure to frost. Likewise, only 13.5% report on the tree height, despite the potentially important vertical temperature gradient.

**Fig. 8:**
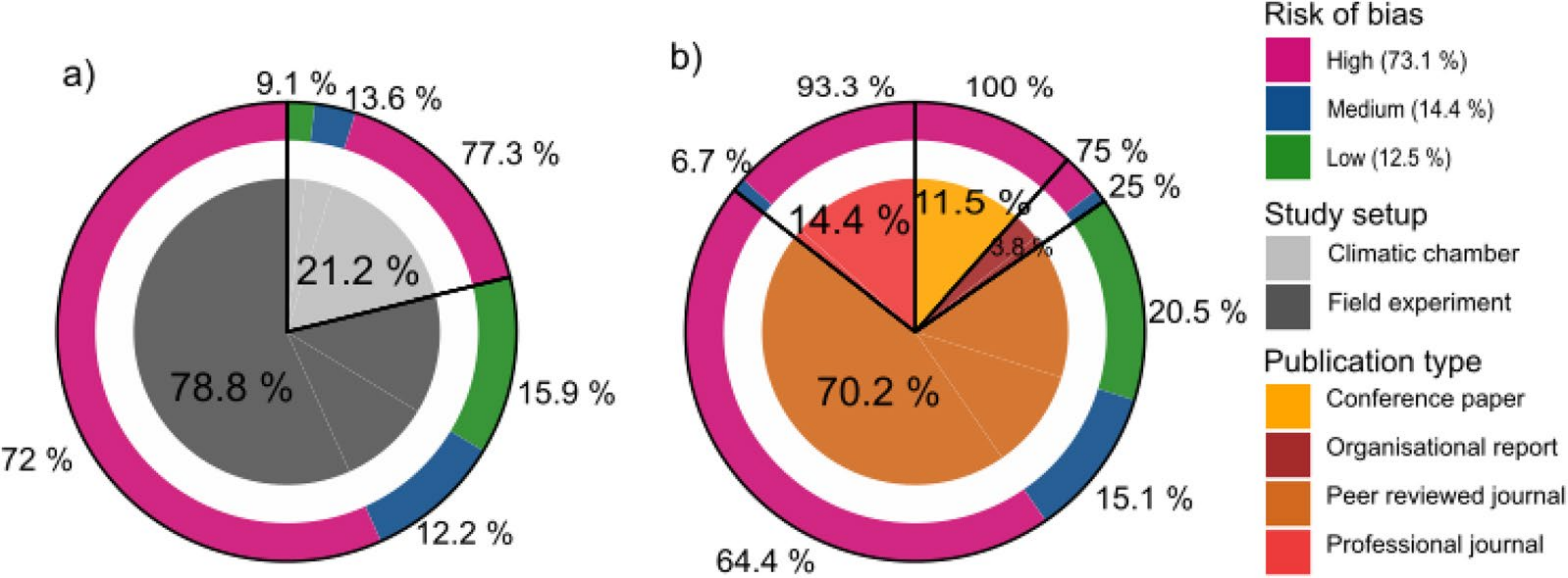
Share of articles judged as having a low, medium or high risk of bias by (**a**) type of study (controlled or field environments) and (**b**) type of publication

For the 104 articles the details on the validity assessment are provided in Additional file 7. With the criteria for risks of biases as defined above, 73.1% of the 104 studies is considered to have a high risk of bias and only 12.5% of the studies were rated to have a low risk for bias, meaning that at most one criterion for a risk of bias was fulfilled. This is illustrated in Fig. 8 where a distinction is made between study setups (Fig. 8a) and types of publications (Fig. 8b), in the inner circles respectively. The major share (78.8%) of the studies were field experiments, while the remainder comprised experiments in controlled environments like cold chambers, tunnels and greenhouses. Surprisingly, the share of low-bias studies was comparatively smaller in the controlled environments (9.1%) than for the field studies (15.9%), where environmental influences cannot be well controlled (see Additional file 8).

Experiments in conference contributions and articles published in practicioners (professional) journals were (nearly) entirely rated as having low validity (Fig. 8b).

**Fig. 9:**
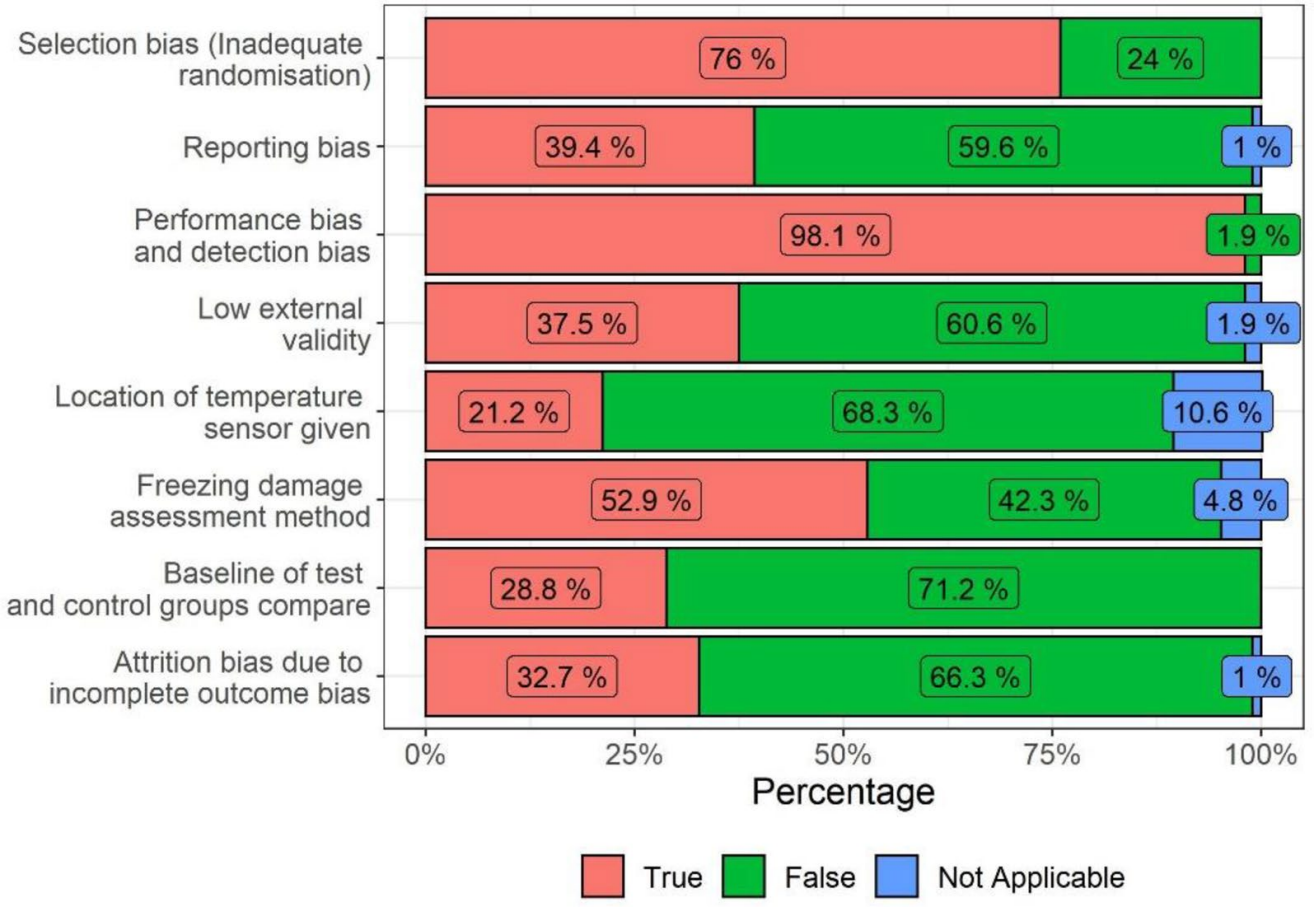
Share of studies with risk of bias-by-bias category

The percentages of studies evaluated as having risks of bias are shown in Fig. 9 per bias type. The “Selection bias” as well as the criteria on “Comparable baselines” refer to biases that result from an unbalanced selection of samples. Most studies did not mention the randomisation of their samples. Studies conducted on selected tree branches in controlled environments should be more suitable for randomisation than those examining entire trees in fields. However, not a single study in controlled experiments reported on the exact way the randomisation was operated, as is the standard in other research fields [44].

In the case of interventions affecting larger spatial extents (e.g., wind machines) a strong spatial separation of the test populations is necessary, which may introduce baseline biases. Adjacent fields have been considered as comparable and risk of bias in this section was only assumed when it was explicitly stated that the control field was not adjacent to the field where the intervention occurred, or in case of other influencing factors like different cultivars.

The “Performance bias” may arise in the absence of blinding and a potential (unconscious) tendency to record higher or lower scores in function of the desired research outcome. In the case of field studies, this is also practically very difficult and only two such studies reported on explicit blinding of the researchers [53, 54]. For example, night temperature data was analysed without knowing which datapoints were collected during wind machine operation.

### Data synthesis

#### Effectiveness of intervention classes

**Fig. 10:**
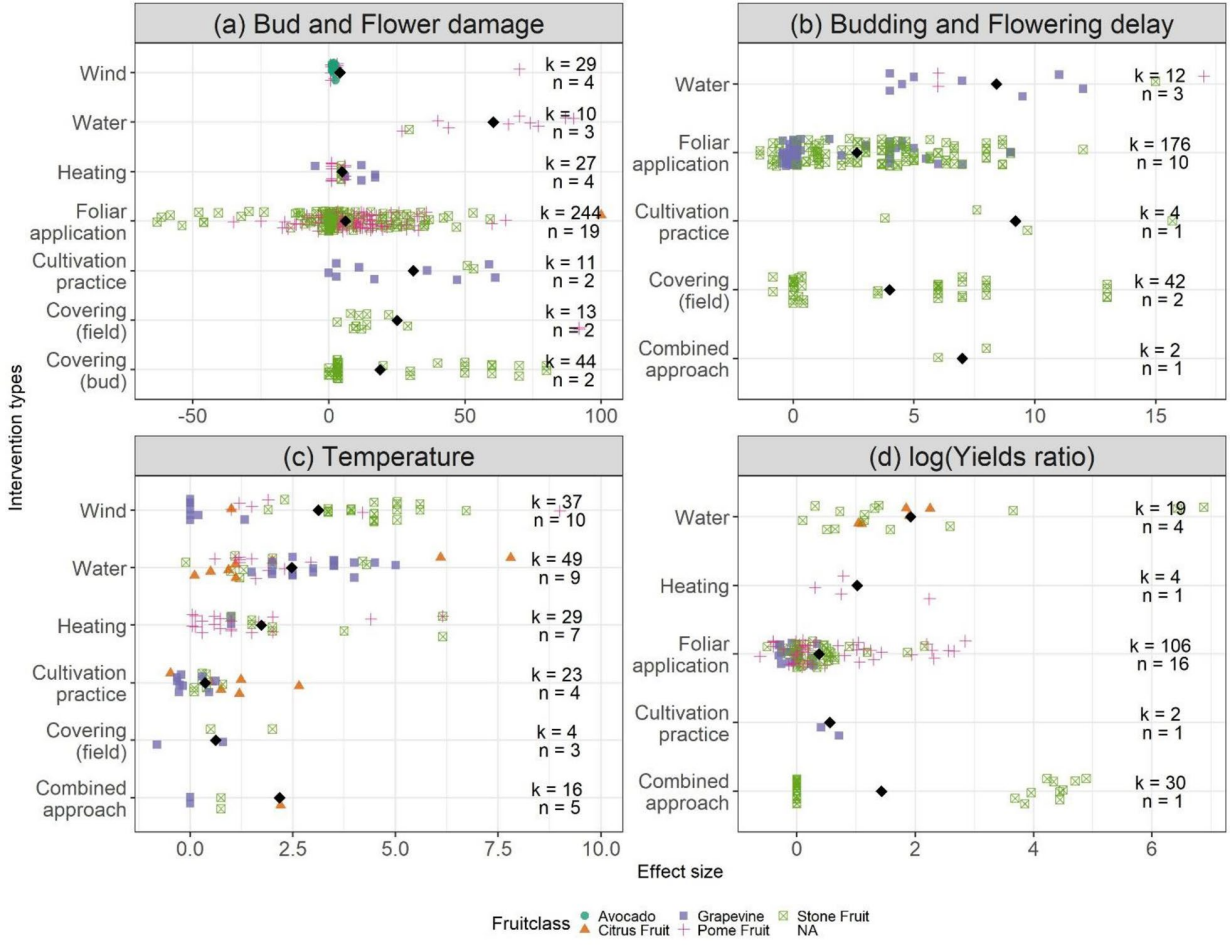
Effectiveness of interventions by outcome variable: **a** the reduction in % damaged flowers and buds, **b** the delay of budding or flowering in days, **c** the maximum reported increase in ambient temperature in °C and **d** the difference in yields expressed as the natural logarithm of the ratio between the control and intervention population. Colour and shape represent the fruit classes, black diamonds represent the mean reported effect by intervention class. Effect size quantities are indicated by ‘k’ and the number of individual articles by ‘n’

Considering all data points, irrespective of the validity, the highest mean bud and flower damage reductions were observed for water-based interventions, followed by the group of cultivation practices (Fig. 10). The average improvement of flower/bud survival was 15.75%. The large group of ‘foliar applications’ appears little effective on average, but several experiments reported above 30% higher survival. Since unsuccessful and even destructive treatments (excessive concentrations, extreme timings) were also included in this comparison, the range of outcomes is wide. Heating systems and tested wind machines seems to have the lowest effectiveness or may have negative effects on flower and bud survival, compared to control populations.

**Fig. 11:**
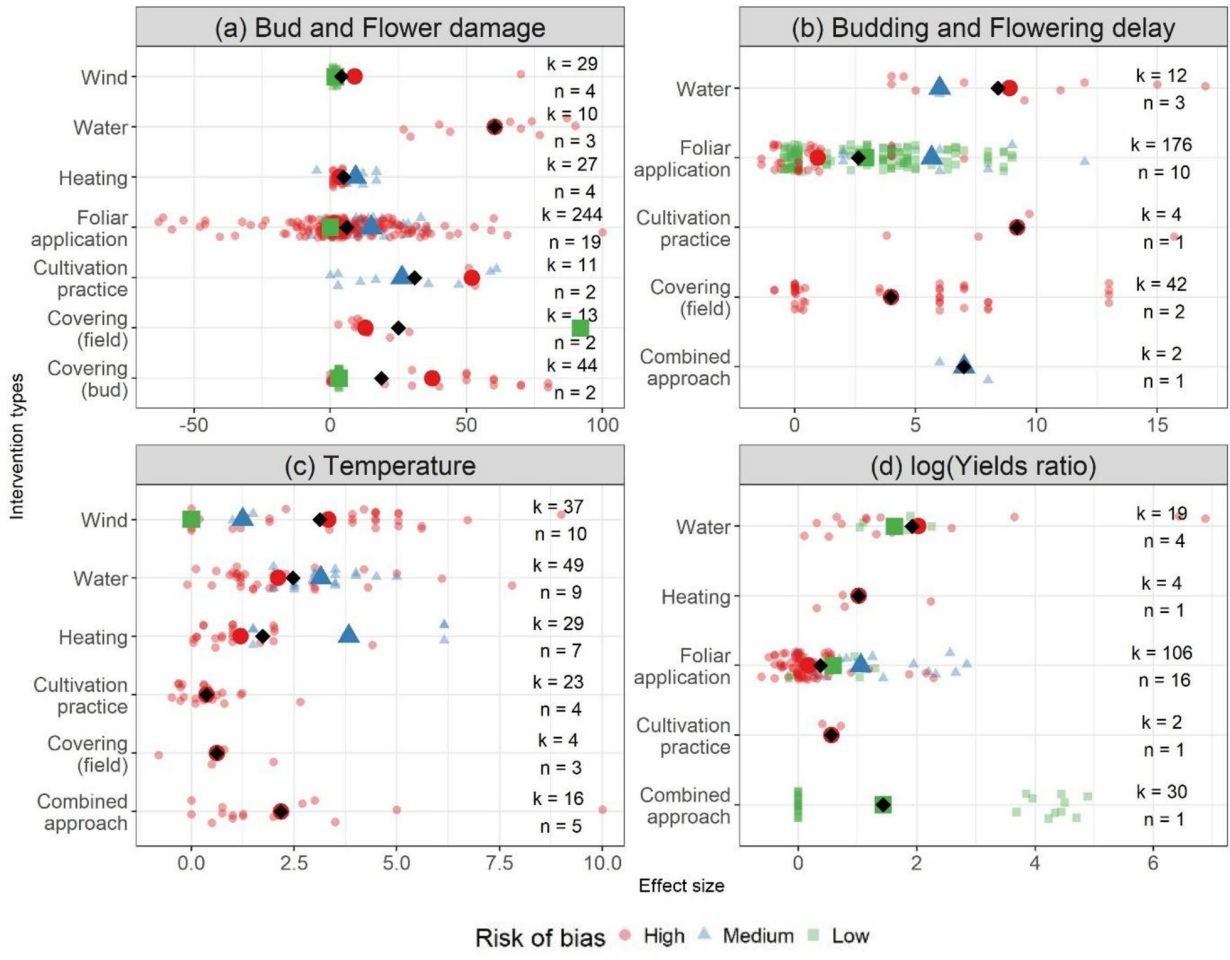
Effectiveness of intervention classes by risk of bias and outcome variables: **a** the reduction in % damaged flowers and buds, **b** the delay of budding or flowering in days, **c** the maximum reported increase in ambient temperature in °C and **d** the difference in yields expressed as the natural logarithm of the ratio of the yield under the intervention and control. Bigger sized symbols represent the mean per Risk of Bias class. Black diamonds represent the mean over all data points. Effect size quantities are indicated by ‘k’ and the number of individual articles by ‘n’

Considering only low risk of bias studies, the comparison does not cover all possible interventions and one single study on field covers emerges as highly effective, while the other techniques do not seem to be effective (Fig. 11).

Delaying of budding or flowering onset is meant to reduce the probability of frosts occurring during the sensitive stage of flowering. Interventions based on water and cultivation practice (mostly pruning techniques) and combined approaches led to increased delays (3–4 days more) compared to most studied foliar applications and installations of tunnels and nets. The mean delay over all techniques was 3.75 days. Here, examining results depending on the risk of bias does not change the conclusions by more than two days.

Only low validity studies reported that wind machine performed best to increase the temperature in orchards and vineyards (Fig. 11). None of the high validity studies report on effective wind machines. The range of temperature increases was large, between 0 and 9 °C. Conventional vertical wind towers outperformed the new horizontal models. Sprinkler systems performed second best and better than combinations of heaters and sprinklers or wind machines as well as heating systems on their own. Alternative systems of other categories failed to exceed a 2.5 °C increase, which may suffice only in case of light frosts. The average increase was 2.1 °C only.

For sprinkler systems the mean value was 0.5, which implies that with the interventions, yields were 1.34 times higher than the yields in the control population. As high and low validity studies reported positive effects, this kind of intervention is worth further investigation.

A Kruskal–Wallis test confirmed significant differences between intervention classes for all the outcome categories (Additional file 1: Table S2) at a significance level p = 0.05. Differences between specific groups are highlighted in the paired Wilcoxon test results per outcome category (Additional file 1: Table S3 – Additional file 1: Table S6). Significant differences were mostly reported for bud and flower damage reductions and the flowering delays. Significant differences in terms of temperature increases and yield ratios were only found between heating and water interventions and between foliar applications and water, respectively.

As pome, stone and citrus fruits and grapevine differ from both biological as managerial perspectives, we distinguish between these classes. A Kruskal–Wallis test confirmed significant differences between at least some fruit classes for all the outcome categories (Additional file 1: Table S7). While the average effects on bud and flower damage were relatively independent of the type of fruit (Fig. 10a, Additional file 1: Table S8), the potential of the interventions meant to delay flowering differed more strongly between stone fruits and grapevine (Additional file 1: Table S9). The increase in ambient temperature should not be dependent on the fruit type for biological reasons, but higher increases were measured in stone fruit orchards (significantly different only compared to pome fruit, Additional file 1: Table S10. Increases in yields were weaker for grapevine than for the other fruits (Additional file 1: Table S11).

#### Conditionality of effectiveness

We hypothesized that the effectiveness of a given measure is dependent on a range of environmental conditions. According to the protocol, we tested four models based on location (elevation, absolute latitude, and their interaction), a soil texture approximation and minimum temperature. The following models are restricted to ‘field experiments’ only and rely on externally retrieved data. The models differed in the defined random factor, which could be either (1) none, (2) the intervention type, (3) the fruit type, or (4) the phenological stage. Given the reporting quality of the collected data, no conclusive statements can be drawn from the explorative regressions. The detailed model results are given in Additional file 1: Table S12 to Additional file 1: Table S15.

**Fig. 12:**
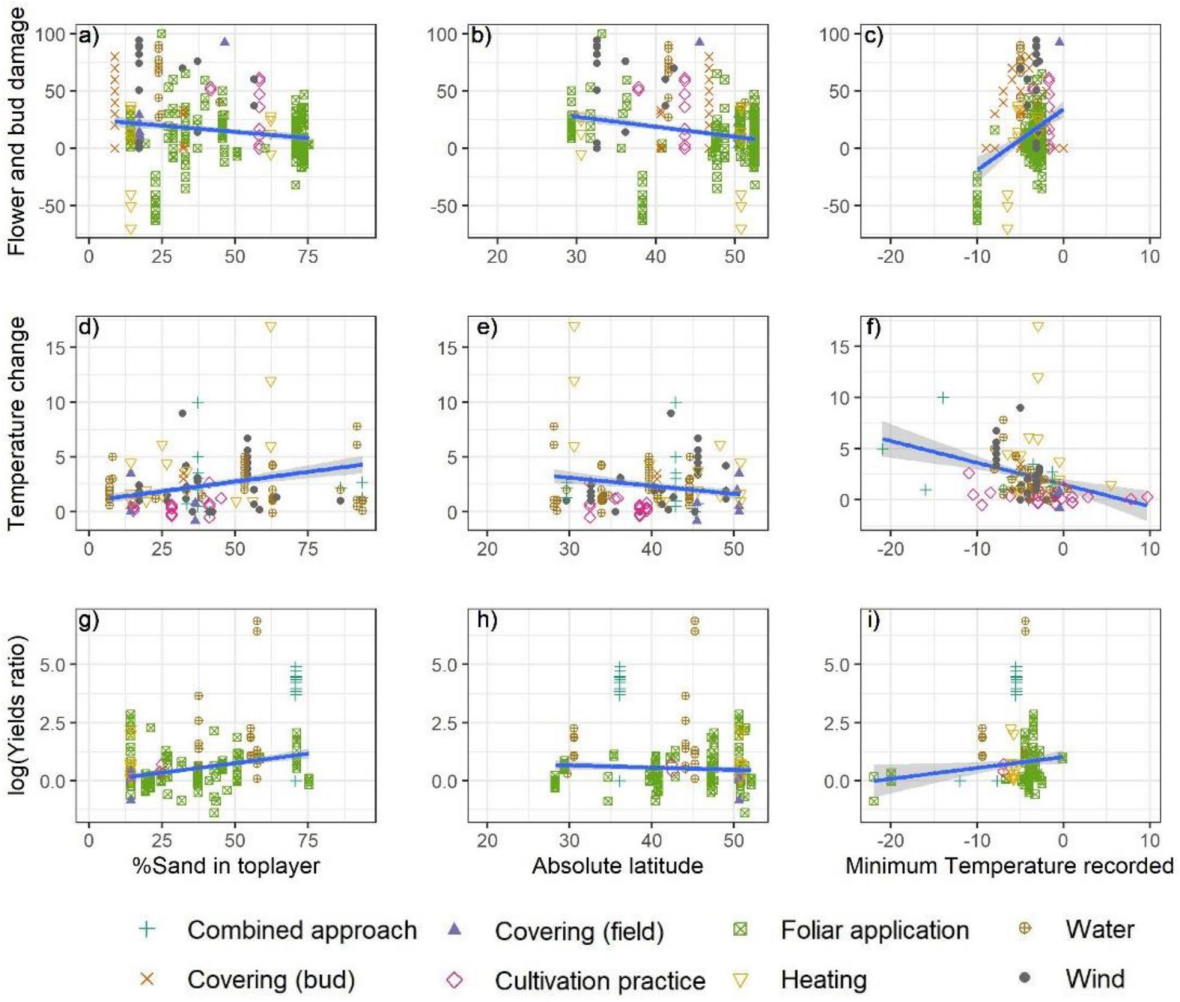
Effect sizes by outcome category (row), intervention class (colour and shape) and by effect modifier: the percentage sand in the top layer (based on SoilGrids Ref. [42], **a**, **d**, **g**), the absolute latitude (**b**, **e**, **h**) and the recorded minimum temperature (**c**, **f**, **i**). Trend lines are derived by linear regression considering all points

In apple orchards, daily temperature ranges were higher and minimum temperature lower on sandy-loamy soils than on clayey soils [39]. The heat capacity and water retention potential of sandy soils are different from soils with a finer texture. However, in function of the employed model and outcome category, relations were both weakly positive and negative. Opposite effects between the impact on bud and flower damage reduction and the other outcomes were reported for the change in ambient temperature, Fig. 12a, d, g).

The latitude could not explain differences in the reported outcomes (Fig. 12b, e, h). Latitude and elevation were considered separately and in interaction, as low latitudes/high altitude locations can be exposed to similar thermal conditions as high latitude/low altitude locations. Effects of the latitude may be correlated with other factors, which are not further investigated, including the economic situation of the country in which a study was conducted, which might influence the means of conducting the study as well as the costs (and quality) of the equipment that was tested.

The severity of the frost (minimum temperature recorded during the experiment) was also expected to pose limits to certain installations more than others. Trend lines were of opposite direction, depending on the outcome measure (Fig. 12c, f, i). In nearly all tested models, the effect was statistically significant (p < 0.001). Based on the available data, which did not allow to detect trends, the highest increases in temperature were documented for temperatures around − 4 °C.

The difference between the models appeared to be influenced by the number of observations. The information on the development stage was only provided in half of the studies. For the interventions aiming at delaying the flowering, only two studies reported on the temperature and the development stage. With R^2^ values of 0.176 (bud and flower damage reduction, 0.458 (temperature), 0.155 (yield ratio), 0.193 (budding and flowering delay), the models have little explanatory power. This suggests that other factors, which could not be tested for, were dominant in determining the effectiveness.

**Fig. 13:**
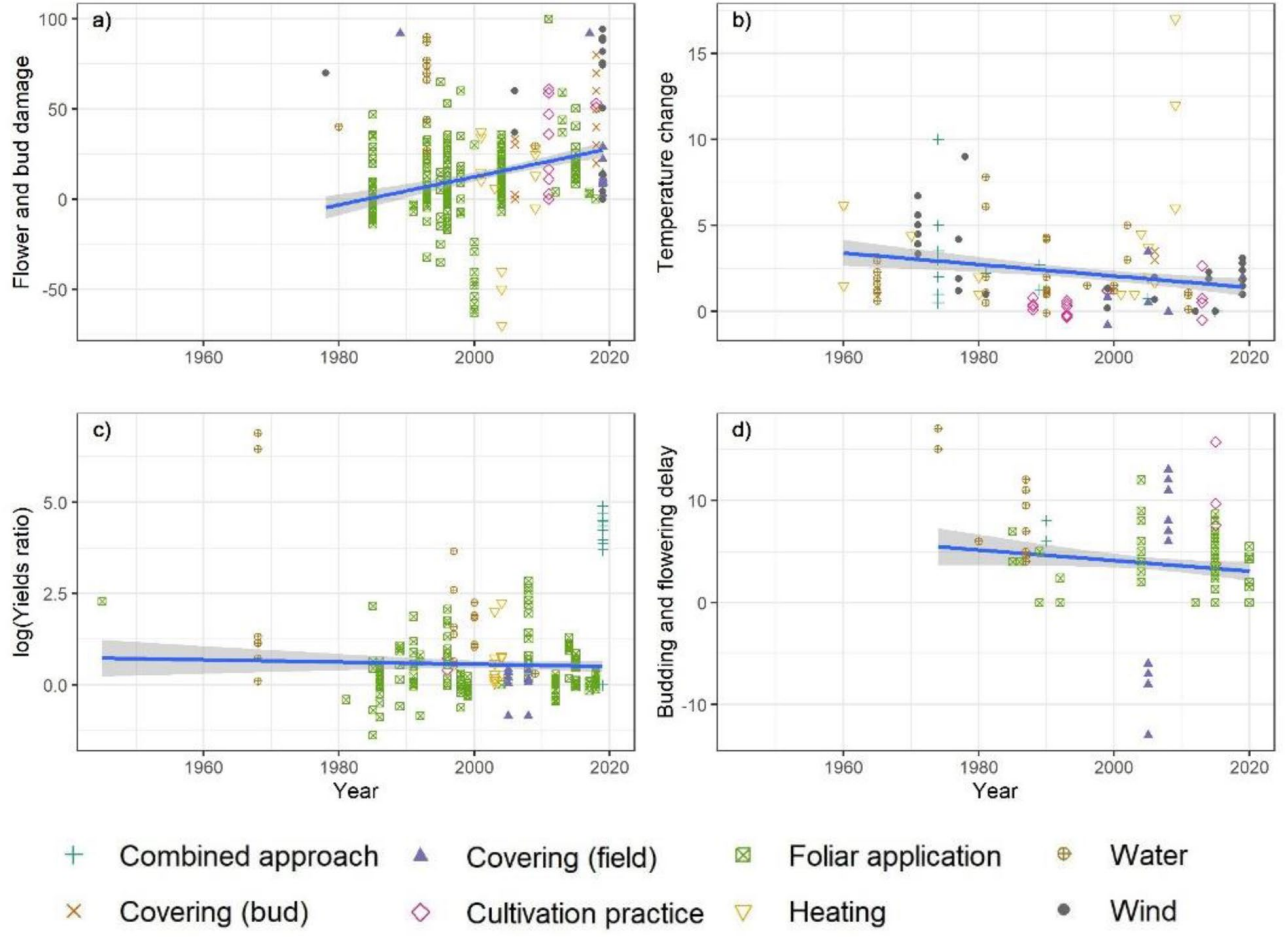
Reported effect sizes by outcome category, intervention class and year. Trend lines are derived by linear regression considering all points

The changes of the reported effectiveness of the tested interventions over time is shown in Fig. 13. The reduction of damage to buds and flowers was reported to increase over time, whereas after 2005 no strong negative outcomes were published anymore. With regard to the other outcomes, the trends in reported effectiveness seemed to be slightly negative. A possible explanation is the growing concern for the resource efficiency of the interventions, like low-volume micro-sprinklers or horizontal wind machines, which do not necessarily deliver the same level of protection as more resource-consuming techniques [17].

#### Sensitivity

The sensitivity of the outcome was assessed with regard to the validity rating of each data point (Fig. 11). It must be noted that, in most cases, there were higher effect sizes in studies with lower validity (Fig. 11). The effect sizes reported by high validity studies are near zero with few exceptions. The other intervention/outcome combinations were not examined in both high and low risk of bias studies. Due to the lack of data and the large dominance of studies with low validity rating, no extensive analysis was possible.

### Review limitations

#### Limitations of review methodology

Within the available resources and given the limited comparability, the review was restricted to studies on interventions that can be measured in terms of yields, temperature increase, budding and flowering delay as well as ambient temperature. It thereby excludes other important practices of (passive) frost protection, including genetic selection or modification, improved rootstocks, increases of frost resistance and antibacterial treatments. The innovativeness of research on spring frost risk management strategies cannot be judged based on the collected evidence base.

Searches were conducted mostly in English and additionally in four other European languages, but no publications in Asian languages (e.g., Chinese) were included. Given the importance of both local fruit production and applied agricultural research in China, a substantial part of the evidence base may have been omitted from the review.

#### Limitations of statistical methods

As only nine studies reported on standard deviations or errors as a measure of precision, no meta-analysis including a quantification of the overall precision, or the heterogeneity of the studies was possible. The definition of sample sizes varied enormously between studies, hindering the estimation of publication biases [55]. Often, the results were more anecdotal evidence with low numbers of repetitions. The statistical metrics are therefore mostly of descriptive nature and the interpretation of the (mixed) linear regression models to answer the question of conditions of effectiveness remains indicative.

Furthermore, due to the range of effect modifiers and the relatively lower number of studies reporting on each category, regression analyses were limited to variables from external data sources, e.g., soil texture, or from meta-data of the study, e.g., the latitude.

#### Limitations of the evidence base

A comparatively low share of full texts could be retrieved, compared to the number of included studies (based on title and abstract). The median year of publication of included studies is 1987. A substantial part of the articles listed in the specialized databases Agricola, FAO Agris and Groene Kennis were not available in digital form and two inquired research centres have disposed of printed copies dating from before 1980. As most of these articles were published in American, German or English practitioner journals, the general conclusions in terms of spatial research gaps are likely to remain valid and the included literature is estimated to be representative, based on a comparative analysis of the abstracts of the missing literature.

Numerous studies from Asian Universities were identified in the queried databases, but the majority was published in Chinese and a good share focussed on topics outside the scope of the review, e.g. crop breeding. This results in a geographic bias. In addition, a focus on high value crops became apparent (grapes, peaches) or high production volumes (apple) rather than commercially less interesting fruits, such as plum.

Certain studies investigated patented products, like the Frostbuster or Frostguard. A minority of articles, especially from earlier years, disclosed their funding source and objectivity. On the other hand, few studies (17.3%) reported on the costs of the interventions, or on other variables which would allow deriving costs. Specialised studies and reports (i.e. [56–62] cover costs, but lack details on effectiveness, resulting in their exclusion from this review. There are also important gaps in the information provided on side effects of the employed techniques, like phytotoxicity [63], waterlogging, noise or reduced fruit quality. Information on the latter attributes was not extracted systematically in this review due to restricted resources.

## Review conclusions

### Implications for policy and management

We investigated the comparative effectiveness of a range of frost protection strategies applied in temperate fruit orchards and vineyards. Almost no studies reported on the precision of the obtained results. Most studies were of low validity and substantial sources of research bias were detected or could not be excluded. The direct findings of the review are therefore of limited transferability in extension or policy.

There seemed to be no evidence, that certain techniques consistently outperform others for all studied outcomes. No recommendation on cost-effectiveness is possible from the gathered information. Since too little information was available on the effect of environmental factors on the effectiveness of the interventions, only limited conclusions could be drawn on whether interventions could be suitable for given locations or fruit businesses. Therefore, the main conclusion for policy and management is the need to enable quality research at dedicated institutes.

### Implications for research

The review is predominantly influenced by research in fruit growing regions in Europe and North America. It was found that not all temperate regions, where fruit is cultivated for commerce is covered by literature. This implies that conclusions are transferable to cultivars and conditions for the covered regions only. Little recent literature was identified to establish theories and correlations guiding the decision making of farmers in regions without local scientific evidence but similar biophysical or topographic conditions as those that were researched. To make better use of published experimental observations, the raw data would be needed, which supports a call for more open data publications.

Over time, the research interest has converged to foliar applications, and to a lesser extent, to wind machines. A focus on grapevine and cherry appears from the evidence map. However, in regions outside Europe, grapevine and cherry are not dominant.

The effectiveness of the studied interventions does not increase with time, except for the reduction of flower or bud damage through foliar sprays.

The inherently heterogeneous nature of perennial fruit production systems and the large variation in experimental methodologies necessitate more rigorous reporting in horticultural research.

From this review the following recommendations for research on frost protection emerged:

1. Document the methodology in a more transparent and explicit manner, e.g., how samples were selected; how outcomes were measured and by how many researchers with which levels of experience;
2. Report the precision of all results using metrics for the reported variables or outcomes, or make raw data available;
3. Publish the experimental protocol describing study sites alongside the article, including information on the soil characteristics, ground cover, the geomorphology, and presence of water bodies;
4. Ensure that control and intervention plots are comparable to each other, and contain sufficient spatial buffer and make this verifiable;
5. Report the meteorological conditions during frost nights (including the minimum temperature, relative humidity and wind speed);
6. Place and shield thermometers or other measuring devices at comparable heights and distances to the intervention and fruit trees;
7. Organise double blinded assessment of yields/damage where the intervention medium is not detectable by assigning this task to a different researcher than the one in charge of the field setup; and
8. Organise triple blind analysis by using random labels for control and intervention outputs

In conclusion, from our findings we suggest the need for a general protocol for research including methodological and reporting standards. Such protocol on (new) techniques for frost protection should be established to ensure comparability across techniques, orchards and locations. These recommendations should facilitate future meta-analysis, allowing to draw more rigorous conclusions.

## Supplementary Information

**Additional file 1: Table S1.** Complete Search strings for the selected libraries with syntax adjusted to advanced search windows or the query URL where applicable. **Table S2.** Kruskal-Wallis rank sum test for intervention classes. **Table S3.** Paired Wilcoxon test p-values for the intervention classes of outcome class 'Bud and flower damage'. **Table S4.** Paired Wilcoxon test p-values for the intervention classes of outcome class ‘Budding and flowering delay’. **Table S5.** Paired Wilcoxon test p-values for the intervention classes of outcome class ‘Temperature’. **Table S6.** Paired Wilcoxon test p-values for the intervention classes of outcome class 'Yields'. **Table S7.** Kruskal-Wallis rank sum test for fruit classes. **Table S8.** Paired Wilcoxon test p-values for the fruit classes of outcome class ‘Bud and flower damage’. **Table S9.** Paired Wilcoxon test p-values for the fruit classes of outcome class ‘Budding and flowering delay’. **Table S10.** Paired Wilcoxon test p-values for the fruit classes of outcome class ‘Temperature’. **Table S11.** Paired Wilcoxon test p-values for the fruit classes of outcome class 'Yields'. **Table S12.** (Mixed) linear model results on effects of location, top layer sand content and minimum temperature during the experiment on Bud and Flower damage reduction**.** **Table S13.** (Mixed) linear model results on effects of location, top layer sand content and minimum temperature during the experiment on Yields. **Table S14.** (Mixed) linear model results on effects of location, top layer sand content and minimum temperature during the experiment on Temperature change. **Table S15.** (Mixed) linear model results on effects of location, top layer sand content and minimum temperature during the experiment on Budding and flowering delay.

**Additional file 2:** Table of all references with reason of exclusion and conflicts.

**Additional file 3:** Coding table with variable names and precoded options.

**Additional file 4: **Data extracted from each study, including metadata and individual study findings, along with other key information such as study location and reporting of effect modifiers.

**Additional file 5: **Table of all eligible articles and the database where an article was found.

**Additional file 6: **Table with unretrievable articles.

**Additional file 7: **Included studies and details on validity assessment.

**Additional file 8: **ROSES form

## Data Availability

This article will be published open access, under the terms of the Creative Commons Attribution 4.0 International License http://creativecommons.org/licenses/by/4.0/). Unrestricted use, distribution, and reproduction in any medium is permitted provided you give appropriate credit to the original author(s) and the source, provide a link to the Creative Commons license, and indicate if changes were made. The Creative Commons Public Domain Dedication waiver (http://creativecommons.org/publicdomain/zero/1.0/) applies to the data made available in this article, unless otherwise stated.
