# Supplementary material for: Strategies for managing spring frost risks in orchards: effectiveness and conditionality—a systematic review
Source: Environ Evid. 2022 Sep 1;11:29. doi: 10.1186/s13750-022-00281-z (PMC11378767; doi:10.1186/s13750-022-00281-z)
Supplement: Supplementary file 1 — Additional file 1: Table S1. Complete Search strings for the selected libraries with syntax adjusted to advanced search windows or the query URL where applicable. Table S2. Kruskal-Wallis rank sum test for intervention classes. Table S3. Paired Wilcoxon test p-values for the intervention classes of outcome class 'Bud and flower damage'. Table S4. Paired Wilcoxon test p-values for the intervention classes of outcome class ‘Budding and flowering delay’. Table S5. Paired Wilcoxon test p-values for the intervention classes of outcome class ‘Temperature’. Table S6. Paired Wilcoxon test p-values for the intervention classes of outcome class 'Yields'. Table S7. Kruskal-Wallis rank sum test for fruit classes. Table S8. Paired Wilcoxon test p-values for the fruit classes of outcome class ‘Bud and flower damage’. Table S9. Paired Wilcoxon test p-values for the fruit classes of outcome class ‘Budding and flowering delay’. Table S10. Paired Wilcoxon test p-values for the fruit classes of outcome class ‘Temperature’. Table S11. Paired Wilcoxon test p-values for the fruit classes of outcome class 'Yields'. Table S12. (Mixed) linear model results on effects of location, top layer sand content and minimum temperature during the experiment on Bud and Flower damage reduction. Table S13. (Mixed) linear model results on effects of location, top layer sand content and minimum temperature during the experiment on Yields. Table S14. (Mixed) linear model results on effects of location, top layer sand content and minimum temperature during the experiment on Temperature change. Table S15. (Mixed) linear model results on effects of location, top layer sand content and minimum temperature during the experiment on Budding and flowering delay. [file 13750_2022_281_MOESM1_ESM.docx]

Additional Table 1 Complete Search strings for the selected libraries with syntax adjusted to advanced search windows or the query URL where applicable

|  | **Language** | **Search String** |
| --- | --- | --- |
| Agris | All | ("Orchard" OR "orchards" OR "fruit tree" OR "fruit trees" OR "pome fruit" OR "pomefruits" OR "stone fruit" OR "stone fruits" OR "hesperidium" OR "hesperidia" OR malus* OR pyrus* OR prunus* OR persea* OR citrus* OR "vitis vinifera" OR "apple" OR "apples" OR "pear" OR "pears" OR "cherry" OR "cherries" OR "peach" OR "peaches" OR "nectarine" OR "nectarines" OR "plum" OR "plums" OR "apricot" OR "apricots" OR "avocado" OR "avocados" OR "lemon" OR "lemons" OR "orange" OR "oranges" OR "grapefruit" OR "grapefruits" OR "mandarine" OR "mandarines" OR "pomelo" OR "pomelos" OR "grape" OR "grapes" OR "vine" OR "vines" OR "vineyard" OR "vineyards") AND (((prevent* OR protect* OR "manage" OR "management" OR damag* OR injur* OR "flowering" OR "flowers" OR "bloom" OR "blooming" OR "blossom*") AND ("frost" OR "frosts" OR freez* OR "cold weather")) OR ((("cold" OR "low* temperature*" ) AND (damag* OR injur* )) OR "Freeze avoidance" OR "antifreeze" OR "anti freeze")) |
| Agricola | All | Search 1  Title = orchard? "fruit tree" "fruit trees'" "pome fruit" "pome fruits" "stone fruit" "stone fruits" hesperidium hesperidia malus? pyrus? prunus? persea? citrus? "vitis vinifera" apple? pear pears cherry cherries peach peaches nectarine? plum plums apricot? avocado? lemon? orange? grapefruit? mandarine? pomelo? grape? vine? vineyard? flowers bloom blooming blossom?  Keyword Anywhere = frost? Freeze?  Subjectcode = 4520  Search 2  Title = orchard? "fruit tree" "fruit trees'" "pome fruit" "pome fruits" "stone fruit" "stone fruits" hesperidium hesperidia malus? pyrus? prunus? persea? citrus? "vitis vinifera" apple? pear pears cherry cherries peach peaches nectarine? plum plums apricot? avocado? lemon? orange? grapefruit? mandarine? pomelo? grape? vine? vineyard? flowers bloom blooming blossom?  Keyword Anywhere = frost? Freeze?  Keyword Anywhere = protect? prevent? |
| CAB abstracts | All | (de:("frost injury" OR "frost protection") AND ab:((orchard? OR "fruit tree" OR "fruit trees'" OR "pome fruit" OR "pome fruits" OR "stone fruit" OR "stone fruits" OR hesperidium OR hesperidia OR malus? OR pyrus? OR prunus? OR persea? OR citrus? OR "vitis vinifera" OR apple? OR pear OR pears OR cherry OR cherries OR peach OR peaches OR nectarine? OR plum OR plums OR apricot? OR avocado? OR lemon? OR orange? OR grapefruit? OR mandarine? OR pomelo? OR grape? OR vine? OR vineyard? OR flowers OR bloom OR blooming OR blossom?)) |
| Groene kennis | English | https://library.wur.nl/WebQuery/gkz-master?gkz=[("orchard](https://library.wur.nl/WebQuery/gkz-master?gkz=%20(%22orchard)" OR "orchards" OR "fruit tree" OR "fruit trees" OR "pome fruit" OR "pome fruits" OR "stone fruit" OR "stone fruits" OR "hesperidium" OR "hesperidia" OR malus* OR pyrus* OR prunus* OR persea* OR citrus* OR "vitis vinifera" OR "apple" OR "apples" OR "pear" OR "pears" OR "cherry" OR "cherries" OR "peach" OR "peaches" OR "nectarine" OR "nectarines" OR "plum" OR "plums" OR "apricot" OR "apricots" OR "avocado" OR "avocados" OR "lemon" OR "lemons" OR "orange" OR "oranges" OR "grapefruit" OR "grapefruits" OR "mandarine" OR "mandarines" OR "pomelo" OR "pomelos" OR "grape" OR "grapes" OR "vine" OR "vines" OR "vineyard" OR "vineyards") AND ( "cold injur*" OR "cold damage*" OR "low* temperature* damage*" OR "low* temperature* injur*" OR "frost" OR "frosts" OR freez* OR "flowering" OR "flowers" OR "bloom" OR "blooming" OR "blossom" OR "blossoming")  &wq_flt=taal&wq_val=en |
|  | German | https://library.wur.nl/WebQuery/gkz-master?gkz=(“Obstbau” OR “Weinbau” OR Obstg*rten OR "Obstb*um*" OR "Kernobst" OR "Steinobst" OR "Hesperidium" OR "Hesperidia" OR "Malus *" OR "Pyrus *" OR "Prunus *" OR "Persea *" OR "Citrus" OR "vitis vinifera" OR "Apfel" OR “Äpfel” OR "Birne" OR “Birnen” OR "Kirsche" OR “Kirschen” OR "Pfirsich" OR “Pfirsiche” OR "Nektarine" OR “Nektarinen” OR "Pflaume" OR “Pflaumen” OR "Aprikose" OR “Aprikosen” OR "Avocado" OR “Avocados” OR "Zitrone" OR “Zitronen” OR "Orange" OR “Orangen” OR "Grapefruit" OR "Mandarine" OR "Mandarinen" OR "Pampelmuse" OR "Pampelmusen" OR “Wein” OR Blüte* OR blüh*) AND ( *frost OR Frost* OR Fröste OR Gefrier* OR Kälte* OR “Schutz” OR “Schaden” OR “Schäden” ) &wq_flt=taal&wq_val=de |
|  | Dutch | https://library.wur.nl/WebQuery/gkz-master?gkz=(“fruitteelt” OR “wijnbouw” OR "Boomgaard" OR "fruitboom" OR "fruitbomen" OR "pomefruit" OR "steenfruit" OR "Hesperidium" OR "Hesperidia" OR Malus* OR Pyrus* OR Prunus* OR Persea* OR Citrus* OR "Appel" OR "Appelen" OR "Peer" OR “Peeren” OR "Kers" OR "Kersen" OR "Perzik" OR "Perziken" OR "Nectarine" OR "Nectarines" OR "Pruim" OR "Pruimen" OR "Abrikoos" OR "Abrikozen" OR "Avocado" OR "Avocados" OR "Citroen" OR "Sinaasappel" OR "Sinaasappelen" OR Grapefruit OR "Mandarijn" OR "Pomelo" OR “Wijn” OR “bloei”) AND (*vorst OR vorst* OR “koud” OR “lentenacht”)  &wq_flt=taal&wq_val=nl |
|  | Dutch | https://library.wur.nl/WebQuery/gkz-master?q=*&wq_flt=trefwoord/dut&wq_val=beschadigingen%20door%20vorst&wq_flt=trefwoord/dut&wq_val=fruitteelt&wq_srt_desc=publicatiejaar |
| Scopus | English | TITLE-ABS-KEY(("orchard$" OR "fruit tree$" OR "pome fruit$" OR "stone fruit$" OR "hesperidium" OR "hesperidia" OR malus* OR pyrus* OR prunus* OR persea* OR citrus* OR "vitis vinifera" OR "apple$" OR "pear" OR "pears" OR "cherry" OR "cherries" OR "peach" OR "peaches" OR "nectarine$" OR "plum" OR "plums" OR "apricot$" OR "avocado$" OR "lemon$" OR "orange$" OR "grapefruit$" OR "mandarine$" OR "pomelo$" OR "grape$" OR "vine$" OR "vineyard$" ) AND ( ( ( prevent* OR protect* OR "manage" OR "management" OR damag* OR injur* OR "flowering" OR "flowers" OR "bloom" OR "blooming" OR "blossom*" ) AND ( "frost" OR "frosts" OR freez* OR "cold weather" ) ) OR ( ( ( "cold" OR "low* temperature*" $ ) W/15 ( damag* OR injur* ) ) OR "Freeze avoidance" OR "antifreeze" OR "anti freeze" ) ) ) |
| Web of Science | English | TS = (( "orchard$" OR "fruit tree$" OR "pome fruit$" OR "stone fruit$" OR "hesperidium" OR "hesperidia" OR malus* OR pyrus* OR prunus* OR persea* OR citrus* OR "vitis vinifera" OR "apple$" OR "pear" OR "pears" OR "cherry" OR "cherries" OR "peach" OR "peaches" OR "nectarine$" OR "plum" OR "plums" OR "apricot$" OR "avocado$" OR "lemon$" OR "orange$" OR "grapefruit$" OR "mandarine$" OR "pomelo$" OR "grape$" OR "vine$" OR "vineyard$") AND (((prevent* OR protect* OR "manage" OR "management" OR damag* OR injur* OR “flowering" OR "flowers" OR "bloom" OR "blooming" OR "blossom*") AND ( "frost" OR "frosts" OR freez* OR "cold weather") ) OR ((("cold” OR "low* temperature*" $) NEAR (damag* OR injur*) ) OR "Freeze avoidance" OR "antifreeze" OR "anti freeze" )) |
| Google Scholar  (protocol) | English | (orchard*\|fruit\|apple*\|pear\|cherry\|peach\| \|nectarine*\|plum\|apricot*\|avocado\|orange*\|mandarine*\|grape*\|vine*) (protect*\| prevent*\| injur*\|damage ) (frost* \|freeze*\| “cold weather” \| “low temperature”) |
| BAIDU Scholar  (protocol) | English | (orchard*\|fruit\|apple*\|pear\|cherry\|peach\| \|nectarine*\|plum\|apricot*\|avocado\|orange*\|mandarine*\|grape*\|vine*) (protect*\| prevent*\| injur*\|damage ) (frost* \|freeze*\| “cold weather” \| “low temperature”) |
| BAIDU Scholar  Adjusted | English | One of the following words:  orchard, fruit, apple, pear, cherry, peach, nectarine, plum, apricot, avocado, orange, mandarine, grape, vine, vineyard  AND  frost protection |

Additional Table 2 Kruskal-Wallis rank sum test for intervention classes.

|  | **Kruskal-Wallis chi-squared** | **Degrees of freedom** | **p-value** |
| --- | --- | --- | --- |
| Bud and flower damage | 52,709 | 7 | 0,000* |
| Budding and flowering delay | 25,981 | 4 | 0,000* |
| Temperature | 25,620 | 6 | 0,000* |
| Yields | 29,524 | 4 | 0,000* |

Additional Table 3 Paired Wilcoxon test p-values for the intervention classes of outcome class 'Bud and flower damage'.

|  | **Covering (bud)** | **Covering (field)** | **Foliar application** | **Heating** | **Cultivation practice** | **Water** |
| --- | --- | --- | --- | --- | --- | --- |
| Covering (field) | 0,087 | - | - | - | - | - |
| Foliar application | 0,027* | 0,024* | - | - | - | - |
| Heating | 0,948 | 0,001* | 0,625 | - | - | - |
| Cultivation practice | 0,697 | 0,786 | 0,034* | 0,078 | - | - |
| Water | 0,001* | 0,004* | 0,000* | 0,000* | 0,027* | - |
| Wind | 0,000* | 0,000* | 0,560 | 0,022* | 0,004* | 0,000* |

Additional Table 4 Paired Wilcoxon test p-values for the intervention classes of outcome class ‘Budding and flowering delay’.

|  | **Combined approach** | **Covering (field)** | **Foliar application** | **Cultivation practice** |
| --- | --- | --- | --- | --- |
| Covering (field) | 0,380 | - | - | - |
| Foliar application | 0,093* | 0,214 | - | - |
| Cultivation practice | 0,950 | 0,093 | 0,046* | - |
| Water | 1,000 | 0,046* | 0,000* | 0,950 |

Additional Table 5 Paired Wilcoxon test p-values for the intervention classes of outcome class ‘Temperature’.

|  | **Combined approach** | **Covering (field)** | **Heating** | **Cultivation practice** | **Water** |
| --- | --- | --- | --- | --- | --- |
| Covering (field) | 0,674 | - | - | - | - |
| Heating | 0,441 | 1 | - | - | - |
| Cultivation practice | 0,674 | 1 | 0,770 | - | - |
| Water | 0,470 | 0,441 | 0,018* | 0,441 | - |
| Wind | 0,371 | 0,450 | 0,076 | 0,441 | 0,371 |

Additional Table 6 Paired Wilcoxon test p-values for the intervention classes of outcome class 'Yields'.

|  | **Combined approach** | **Foliar application** | **Heating** | **Cultivation practice** |
| --- | --- | --- | --- | --- |
| Foliar application | 0,347 | - | - | - |
| Heating | 0,347 | 0,096 | - | - |
| Cultivation practice | 0,439 | 0,347 | 0,533 | - |
| Water | 0,075 | 0,000* | 0,405 | 0,347 |

Additional Table 7 Kruskal-Wallis rank sum test for fruit classes

|  | **Kruskal-Wallis chi-squared** | **Degrees of freedom** | **p-value** |
| --- | --- | --- | --- |
| Bud and flower damage | 14,278 | 4 | 0,006* |
| Budding and flowering delay | 31,628 | 2 | 0,000* |
| Temperature | 87,167 | 3 | 0,033* |
| Yields | 33,181 | 3 | 0,000* |

Additional Table 8 Paired Wilcoxon test p-values for the fruit classes of outcome class ‘Bud and flower damage’.

|  | **Avocado** | **Citrus Fruit** | **Grapevine** | **Pome Fruit** |
| --- | --- | --- | --- | --- |
| Citrus Fruit | 0,194 | - | - | - |
| Grapevine | 0,426 | 0,194 | - | - |
| Pome Fruit | 0,027* | 0,194 | 0,426 | - |
| Stone Fruit | 0,287 | 0,194 | 0,668 | 0,027* |

Additional Table 9 Paired Wilcoxon test p-values for the fruit classes of outcome class ‘Budding and flowering delay’.

|  | **Grapevine** | **Pome Fruit** |
| --- | --- | --- |
| Pome Fruit | 0,018* | - |
| Stone Fruit | 0,000* | 0,042* |

Additional Table 10 Paired Wilcoxon test p-values for the fruit classes of outcome class ‘Temperature’.

|  | **Citrus Fruit** | **Grapevine** | **Pome Fruit** |
| --- | --- | --- | --- |
| Grapevine | 0,801 | - | - |
| Pome Fruit | 0,801 | 0,632 | - |
| Stone Fruit | 0,470 | 0,113 | 0,037* |

Additional Table 11 Paired Wilcoxon test p-values for the fruit classes of outcome class 'Yields'.

|  | **Citrus Fruit** | **Grapevine** | **Pome Fruit** |
| --- | --- | --- | --- |
| Grapevine | 0,001* | - | - |
| Pome Fruit | 0,024* | 0,000* | - |
| Stone Fruit | 0,044* | 0,000* | 0,634 |

Additional Table 12 (Mixed) linear model results on effects of location, top layer sand content and minimum temperature during the experiment on Bud and Flower damage reduction

========================================================================
 Dependent variable: Bud and Flower damage reduction
 --------------------------------------------------
 OLS linear mixed-effects
 (1) (2) (3) (4)
 ----------------------------------------------------------------------
 Elevation 0.079** 0.054 0.063* 0.095
 (0.028) (0.040) (0.029) (0.051)

 Absolute latitude 0.853* 0.550 0.292 0.933*
 (0.329) (0.517) (0.400) (0.440)

 % Sand in top layer 0.145* -0.024 -0.174* -0.556***
 (0.068) (0.099) (0.070) (0.113)

 Minimum temperature 6.784*** 3.942* 6.877*** 1.979
 (1.288) (1.658) (1.345) (1.066)

 Elevation:Absolute latitude-0.002** -0.001 -0.002* -0.002
 (0.001) (0.001) (0.001) (0.001)

 Constant 2.784 4.788 28.366 9.216
 (13.047) (21.331) (17.460) (18.952)
 ----------------------------------------------------------------------
 Observations 149 149 149 214
 R2 0.176
 Adjusted R2 0.147
 Log Likelihood -637.576 -640.382 -935.631
 Akaike Inf. Crit. 1291.153 1296.765 1887.262
 Bayesian Inf. Crit. 1315.184 1320.796 1914.190
 Residual Std. Error 16.905 (df = 143)
 F Statistic 6.113*** (df = 5; 143)
 ======================================================================
 Note: *p<0.05; **p<0.01; ***p<0.001

Additional Table 13 (Mixed) linear model results on effects of location, top layer sand content and minimum temperature during the experiment on Yields

=====================================================================
 Dependent variable: Yields
 -------------------------------------------------
 OLS linear mixed-effects
 (1) (2) (3) (4)
 ---------------------------------------------------------------------
 Elevation -0.004 -0.006** -0.003 -0.006
 (0.002) (0.002) (0.003) (0.004)

 Absolute latitude -0.050 -0.083* -0.074 -0.091*
 (0.037) (0.037) (0.059) (0.043)

 % Sand in top layer 0.022** -0.023* 0.007 0.004
 (0.008) (0.009) (0.012) (0.011)

 Minimum temperature 0.189** 0.457*** 0.251*** 0.045
 (0.061) (0.062) (0.064) (0.067)

 Elevation:Absolute lat. 0.0001 0.0001* 0.0001 0.0001
 (0.0001) (0.00005) (0.0001) (0.0001)

 Constant 3.164 8.938*** 4.822 5.514*
 (1.834) (2.174) (2.841) (2.462)

 ---------------------------------------------------------------------
 Observations 120 120 120 64
 R2 0.155
 Adjusted R2 0.118
 Log Likelihood -214.700 -232.636 -113.304
 Akaike Inf. Crit. 445.400 481.272 242.609
 Bayesian Inf. Crit. 467.700 503.572 259.880
 Residual Std. Error 1.402 (df = 114)
 F Statistic 4.177** (df = 5; 114)
 =====================================================================
 Note: *p<0.05; **p<0.01; ***p<0.001

Additional Table 14 (Mixed) linear model results on effects of location, top layer sand content and minimum temperature during the experiment on Temperature change

===============================================================
 Dependent variable: Temperature change
 -------------------------------------------
 OLS linear mixed-effects
 (1) (2) (3)
 ---------------------------------------------------------------
 Elevation -0.026*** -0.026*** -0.023***
 (0.004) (0.005) (0.004)

 Absolute latitude -0.051 -0.040 -0.060
 (0.042) (0.044) (0.040)

 % Sand in top layer 0.020* 0.021* 0.026*
 (0.009) (0.010) (0.010)

 Minimum temperature -0.261*** -0.251*** -0.213***
 (0.049) (0.051) (0.048)

 Elevation:Absolute lat. 0.001*** 0.001*** 0.001***
 (0.0001) (0.0001) (0.0001)

 Constant 1.090 0.574 1.304
 (1.856) (1.956) (1.774)

 ---------------------------------------------------------------
 Observations 120 120 117
 R2 0.458
 Adjusted R2 0.434
 Log Likelihood -243.590 -231.327
 Akaike Inf. Crit. 503.180 478.654
 Bayesian Inf. Crit. 525.479 500.752
 Residual Std. Error 1.522 (df = 114)
 F Statistic 19.250*** (df = 5; 114)
 ===============================================================
 Note: *p<0.05; **p<0.01; ***p<0.001

Additional Table 15 (Mixed) linear model results on effects of location, top layer sand content and minimum temperature during the experiment on Budding and flowering delay

===============================================================
 Dependent variable: Budding and Flowering delay
 -------------------------------------------
 OLS linear mixed-effects
 (1) (2) (3)
 ---------------------------------------------------------------
 Elevation 0.009*** 0.004 0.009***
 (0.002) (0.003) (0.002)

 Absolute latitude 0.283*** 0.044 0.265**
 (0.070) (0.132) (0.086)

 % Sand in top layer -0.080* -0.061 -0.078*
 (0.031) (0.042) (0.032)

 Elevation:Absolute latitude -0.0002*** -0.0001 -0.0002***
 (0.00005) (0.0001) (0.0001)

 Constant -8.069* 4.694 -5.426
 (3.188) (5.267) (4.470)
 ---------------------------------------------------------------
 Observations 203 203 203
 R2 0.193
 Adjusted R2 0.177
 Log Likelihood -518.988 -551.327
 Akaike Inf. Crit. 1051.977 1116.653
 Bayesian Inf. Crit. 1075.169 1139.846
 Residual Std. Error 3.378 (df = 198)
 F Statistic 11.863*** (df = 4; 198)
 ===============================================================
 Note: *p<0.05; **p<0.01; ***p<0.001
